# Gluon PDF constraints from the ratio of forward heavy-quark production at the LHC at $\sqrt{S}=7$ and 13 TeV

**DOI:** 10.1140/epjc/s10052-015-3814-x

**Published:** 2015-12-22

**Authors:** Matteo Cacciari, Michelangelo L. Mangano, Paolo Nason

**Affiliations:** Université Paris Diderot, 75013 Paris, France; Sorbonne Universités, UPMC Univ Paris 06, UMR 7589, LPTHE, 75005 Paris, France; UMR 7589, LPTHE, CNRS, 75005 Paris, France; PH-TH, CERN, 1211 Geneva, Switzerland; INFN, Sezione di Milano Bicocca, Piazza della Scienza 3, 20126 Milan, Italy

## Abstract

We discuss production of charm and bottom quarks at forward rapidity in *pp* collisions at the LHC, updating the QCD predictions for the run at $\sqrt{S}=13$ TeV. We show that, while the absolute rates suffer from large theoretical systematics, dominated by scale uncertainties, the increase relative to the rates precisely measured at 7 TeV can be predicted with an accuracy of a few percent, sufficient to highlight the sensitivity to the gluon distribution function.

## Introduction

**Fig. 1:**
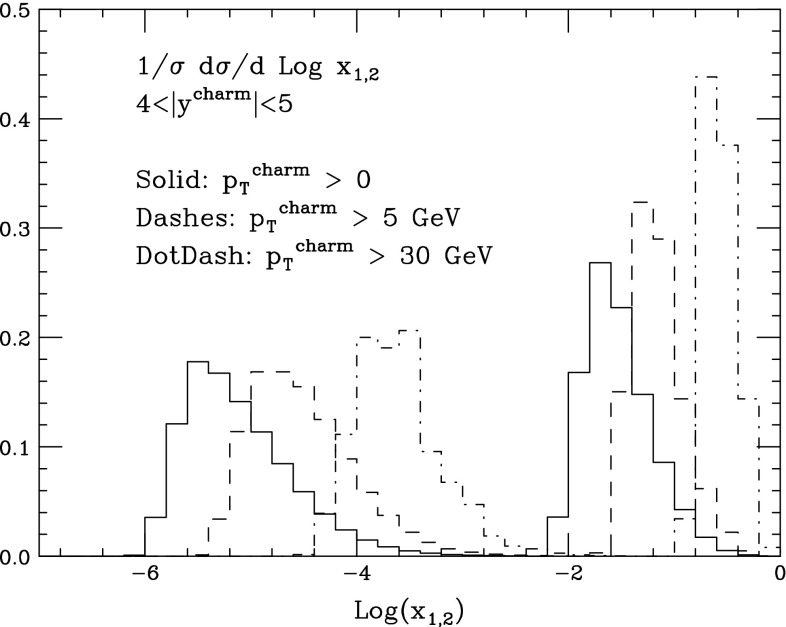
Distribution of partonic fractional momentum *x* in charm pair production, at LO in *pp* collisions at $\sqrt{S}=13$ TeV. Events are required to have a charm quark in the forward region, $4<\vert y \vert <5$. The different curves correspond to various minimum thresholds for the quark $p_T$, namely 0, 5 and 30 GeV. All curves are equally normalised

Measurements of heavy-quark ($Q=c,b$) production rates from all LHC experiments during Run 1 [1–27] have shown agreement, within the estimated systematics, between data and theoretical predictions [28–31]. These systematics are typically dominated by theoretical uncertainties, which are very large: the renormalisation and factorisation scale dependence, the value of the heavy-quark mass, and, to a lesser extent, the uncertainties of the parton distribution functions (PDFs). In particular, the scale uncertainty at small transverse momentum $p_T$ (namely $p_T\sim m_Q$, where $m_Q$ is the heavy-quark mass) reaches values in the range of $\sim$100 % in the case of the charm quark, and of $\sim$50 % for the bottom quark. This situation prevents the use of heavy quarks for precision measurements. This is frustrating, since the experiments have proven their ability to measure charm and bottom quarks in regions of small $p_T$, as well as of large rapidity, where production properties probe very interesting dynamical regimes, and are sensitive to the gluon PDFs in both the small- and the large-*x* regions.^1^ This is shown for example in Fig. 1, where we plot, at leading order (LO) in QCD, the distribution of partonic *x* values in final states of *pp* collisions at $\sqrt{S}=13$ TeV where a charm quark is produced in the rapidity range $4<\vert y \vert < 5$. For small-$p_T$ production, one probes *x* values in the region $x\lesssim 10^{-5}$, while for $p_T \gtrsim 30$ GeV one probes $x\gtrsim 0.2$.

In Ref. [31] it was suggested to use, for PDF studies, only the information encoded in the shape of heavy-quark differential distributions, since this is more stable with respect to theoretical uncertainties. In this work, we show that the predictivity of the theoretical calculations can be improved by considering appropriate observables, exploiting the future availability of LHC results at different energies, namely $\sqrt{S}=13$ TeV in addition to the lower energy data already available at 7 and 8 TeV.^2^ The main idea, introduced in a general context in Ref. [32], is to consider ratios of kinematical distributions of heavy quarks—e.g. inclusive $p_T$ and *y* spectra—at different energies.^3^ For a given parton-level kinematics, the structure of the logarithmic dependence on the renormalisation and factorisation scales is independent of the beam energy, since it just depends on the partonic momenta. This allows one, when studying the scale dependence of the cross-section ratios, to correlate the scale choice made at the two energies, leading to a major reduction in the sensitivity to the scale variation. On the other hand, the same $(p_T,y)$ kinematics selects initial-state partons with different values of *x*, since at fixed *y* we have $x\propto p_T/\sqrt{S}$. This means that, even though the choice of PDF at the two energies must be correlated, PDFs having different *x* dependence will predict different values for the cross-section ratios, leading to possibly useful constraints for the PDF fits. Other parameters such as the heavy-quark mass, or, when it comes to the complete prediction of realistic final states, fragmentation fractions to specific hadrons, fragmentation functions and decay branching ratios, are also fully correlated at different energies and lead to totally negligible systematics in the cross-section ratios.

The main outcome of these considerations is that, on the basis of the measurements already performed at 7 TeV, one can predict with much greater accuracy the cross sections at 13 TeV, and possibly be sensitive to the (mostly gluon) PDF in regions where it is not yet well constrained by data. In the rest of the paper we analyse more quantitatively these statements, focusing on the potential of the LHCb and, partly, ALICE experiments to combine results from the forthcoming 13 TeV runs and previous 7 or 8 TeV runs. As part of this work, we also update to 13 TeV the complete predictions for absolute cross sections presented in Ref. [28]. We refer to this work for the detailed description of the general framework of our calculations, and for the earlier literature.

## General considerations

**Fig. 2:**
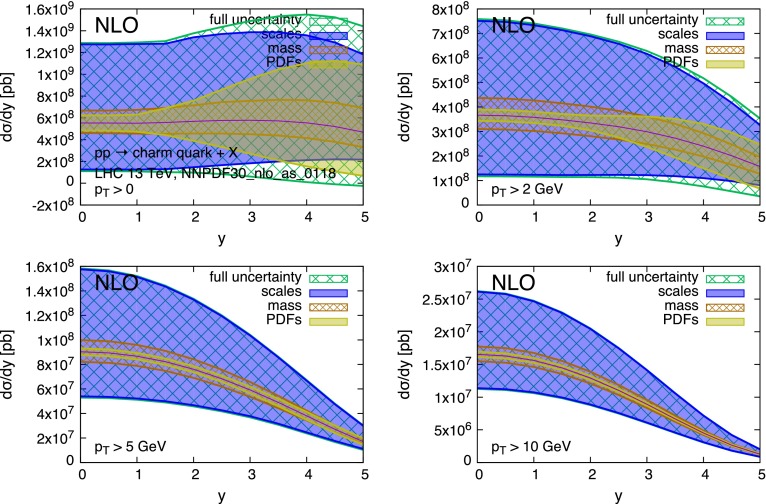
Charm quark rapidity distributions at $\sqrt{S}=13$ TeV

**Fig. 3:**
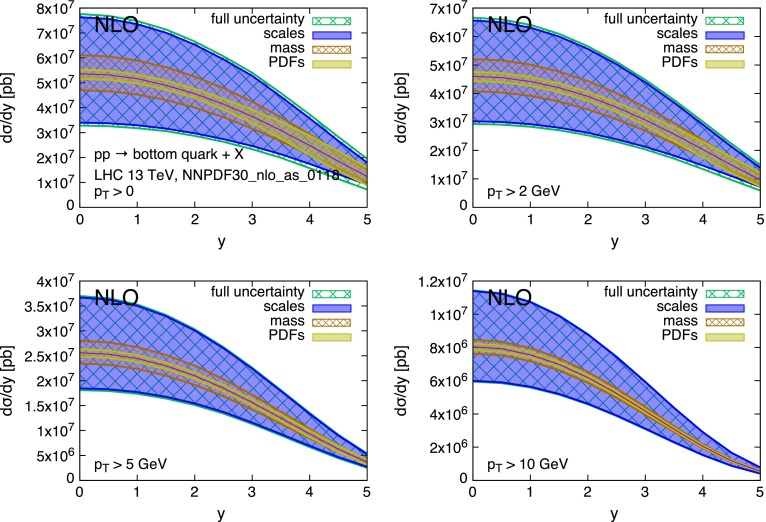
Bottom quark rapidity distributions at $\sqrt{S}=13$ TeV

**Fig. 4:**
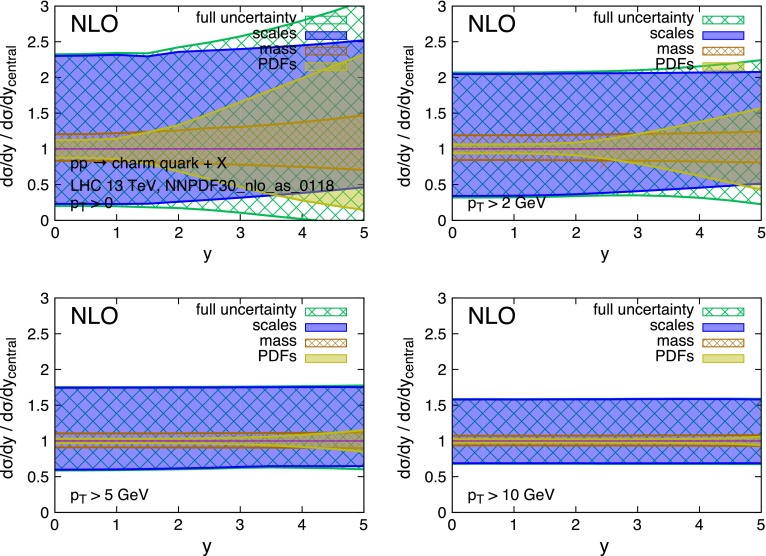
Charm quark rapidity distributions at $\sqrt{S}=13$ TeV, normalised to the central theoretical prediction

**Fig. 5:**
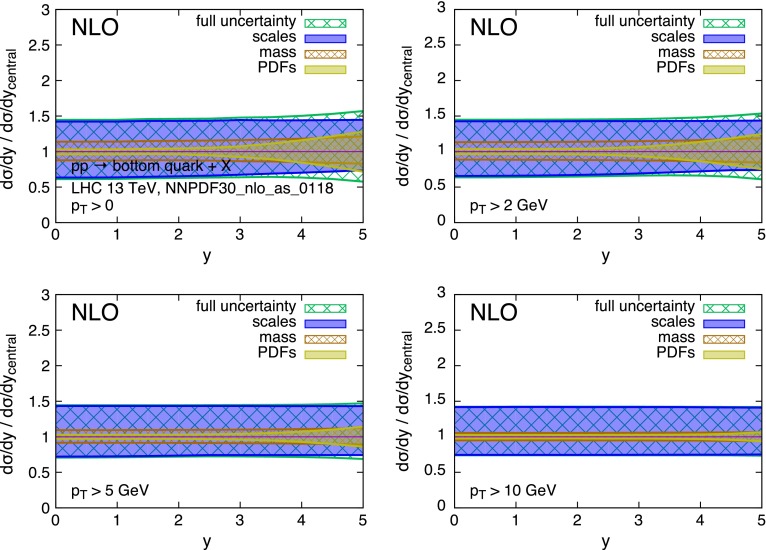
Bottom quark rapidity distributions at $\sqrt{S}=13$ TeV, normalised to the central theoretical prediction

The strong scale dependence in charm and bottom pair production is mostly the consequence of the large corrections [35–37] at the next-to-leading order (NLO), and possibly beyond. This is due in part to the intrinsically large value of $\alpha _S(\mu )$ at the relevant scales $\mu \sim m_Q$, and in part to the emergence of new processes at $\mathcal{O}(\alpha _S^3)$. The large uncertainty can be mitigated in the regime of $p_T \gg m_Q$, where the dominant higher-order contributions have a universal logarithmic behaviour that allows for their resummation [38]. At lower $p_T$ values, where we can only rely on the fixed-order NLO QCD calculation,^4^ the scale dependence reaches values in the range of $\sim$100 % in the case of the charm quark, and of $\sim$50 % for the bottom quark. This situation is shown in more detail in Figs. 2 and 3. These show, for *pp* collisions at $\sqrt{S} = 13$ TeV,^5^ the production cross section $d\sigma /dy$ for charm and bottom quarks, calculated at the NLO. The scale uncertainty is estimated using, as usual, the envelope of the 7-point scale variation:

1 $$\begin{aligned} (\mu _R,\mu _F)= & {} [(1/2,1/2),(1,1/2),(1/2,1),\nonumber \\&(1,1),(1,2),(2,1)]\times m_T, \end{aligned}$$

with $m_T = \sqrt{m_Q^2 + p_T^2}$. The scale uncertainty easily dwarves all other sources of uncertainties, namely the heavy-quark mass value and PDFs. This is especially true at small transverse momentum $p_T$ and central rapidity *y*. Figures 4 and 5 show the same data but normalised to the central theoretical prediction. The relative size of the various uncertainties can be better appreciated here.

As anticipated in the Introduction, we shall consider here the ratio of differential distributions between different $\sqrt{S}$ values. In particular, we focus on the rapidity spectra and define

2 $$\begin{aligned} R(y) \equiv \frac{\mathrm{d}\sigma /\mathrm{d}y \,(13\,\mathrm {TeV})}{\mathrm{d}\sigma /\mathrm{d}y \,(7\,\mathrm {TeV})} \end{aligned}$$

We argued before that, in the evaluation of the scale uncertainty, it is justified to correlate the scale choices made at the two energies. This is because the scale of the process is in fact independent of the collision energy, and it is mainly a function of the process transverse kinematics.

**Fig. 6:**
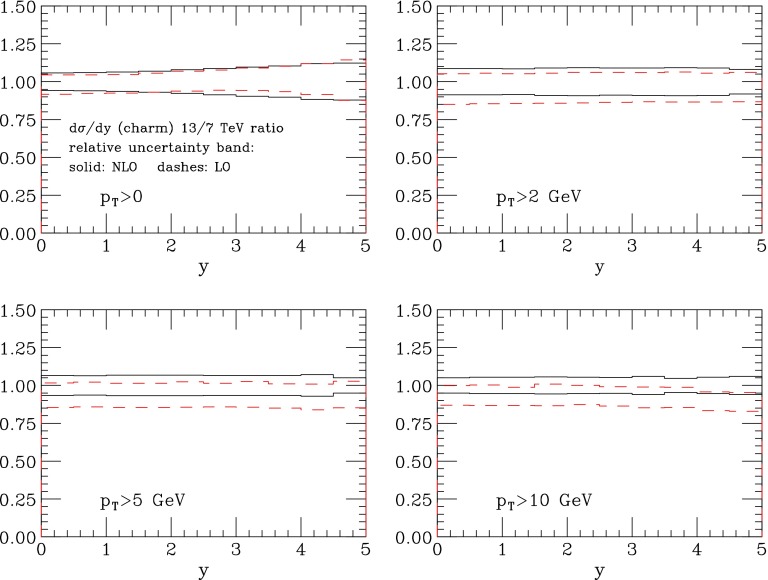
Relative uncertainty in the ratios of the charm rapidity differential distribution between 13 and 7 TeV, for different $p_T$ ranges. The *solid* (*dashed*) *lines* represent the spread of scale dependence at NLO (LO), relative to the centre of the NLO band

Higher-order corrections not directly related to the regularisation process could be more or less enhanced by a higher-energy regime. This is in principle the case of the so-called small-*x* logarithmic effects [41, 42]. However, the evolution in energy from 7 to 13 TeV is not sufficient to expose large effects. As a sanity check of this statement, we compare the NLO predictions for the *R*(*y*) ratios to the LO ($R^\mathrm{LO}(y)$) ones. Defining $R^{(\mathrm LO)}_\mathrm{max,\mathrm min}(y)$ as the upper and lower extremes in the envelope of $R^{(\mathrm LO)}(y)$ values obtained by scanning over the scales choices given in Eq. (1), and defining the centre of the NLO envelope by $R_{0}(y)=(R_\mathrm{max}(y)+R_\mathrm{min}(y))/2$, Fig. 6 shows the following distributions, for different charm $p_T$ thresholds ($p_T>0, \, 2,\, 5, \, 10$ GeV):

$$\begin{aligned} \mathrm {Solid~lines}{:} \quad \frac{R_\mathrm{max}(y)}{R_0(y)} \quad \mathrm {and} \quad \frac{R_\mathrm{min}(y)}{R_0(y)} \; , \nonumber \\ \mathrm {Dahed~lines}{:} \quad \frac{R^\mathrm{LO}_\mathrm{max}(y)}{R_0(y)} \quad \mathrm {and} \quad \frac{R^\mathrm{LO}_\mathrm{min}(y)}{R_0(y)} \;. \end{aligned}$$

Figure 6 shows that our assumption, that a standard scale variation with the same choice of the scales in the numerator and denominator yields a reasonable estimate of the perturbative uncertainty, is verified when going from a LO to an NLO calculation. In fact, the difference between the LO and NLO result is always well below the error that is estimated using the scale variation in the LO result. In other words, *R*(*y*) is very stable with respect to radiative corrections, in spite of the fact that the NLO leads to a *K* factor larger than 2 for the absolute rates. We also notice that the NLO uncertainty on *R*(*y*) is only mildly reduced with respect to the LO one, and mostly for the higher $p_T$ values. The effect is minimal since, as is well known for charm production, the absolute scale uncertainty at NLO is of the same order of magnitude as at LO. Nevertheless, we see confirmed the expectation that the scale dependence of the ratio is much smaller than that of the rates at the individual energies, being in the 5–10 % range, depending on $p_T$ and *y*.

## Results

We show in this section the complete determination of the ratio systematics, including the other sources of uncertainty. From now on we only consider the NLO results for *R*(*y*). The ratios *R*(*y*) are calculated using at both centre-of-mass energies the same renormalisation/factorisation scales, the same mass values and the same PDF set members. As a reference PDF set for this study, we use the recent NNPDF30$\_$nlo$\_$as$\_$0118 [43], as implemented in LHAPDF [44].

**Fig. 7:**
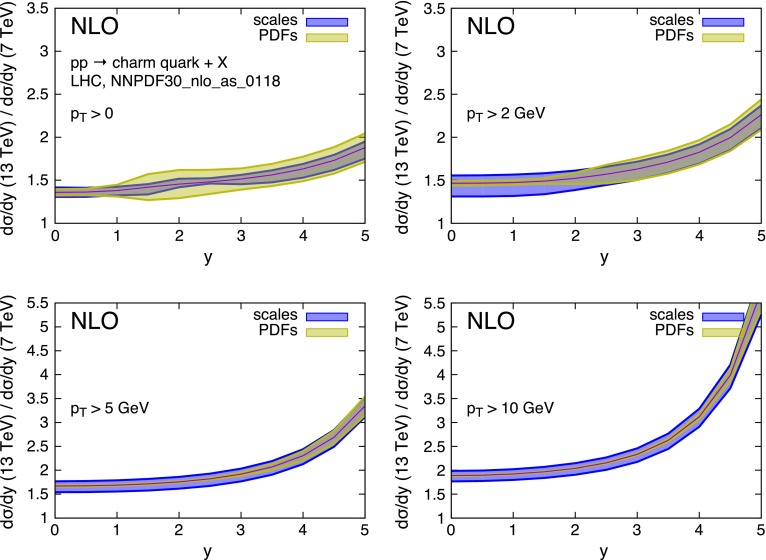
Ratio of charm quark rapidity distributions in *pp* collisions at $\sqrt{S}=13$ TeV and $\sqrt{S}=7$ TeV collisions in the LHC

**Fig. 8:**
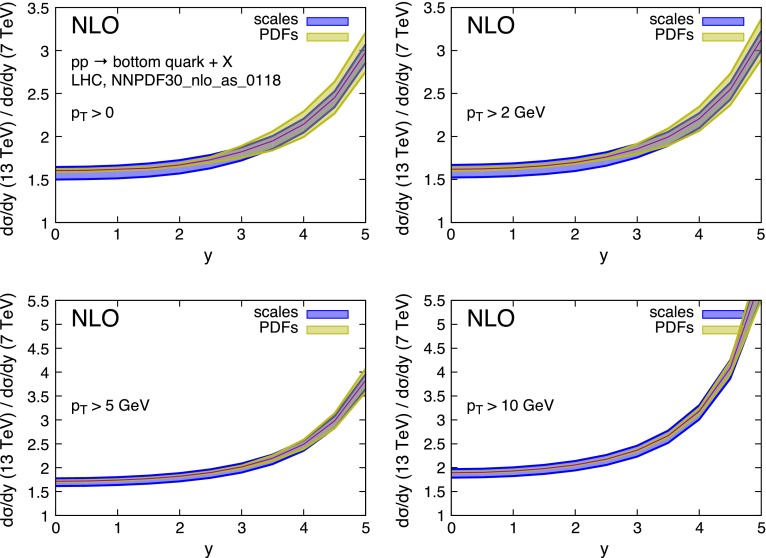
Ratio of bottom quark rapidity distributions in *pp* collisions at $\sqrt{S}=13$ TeV and $\sqrt{S}=7$ TeV collisions in the LHC

The *R*(*y*) distributions, for various $p_T$ thresholds, are shown in Figs. 7 and 8. Thanks to the important suppression of the scale dependence, the overall uncertainties are greatly reduced and are now of order 10 % rather than 50–100 %. More importantly, the PDF uncertainty can now become the dominant one,^6^ if one considers production at sufficiently low transverse momentum and sufficiently forward rapidity.

**Fig. 9:**
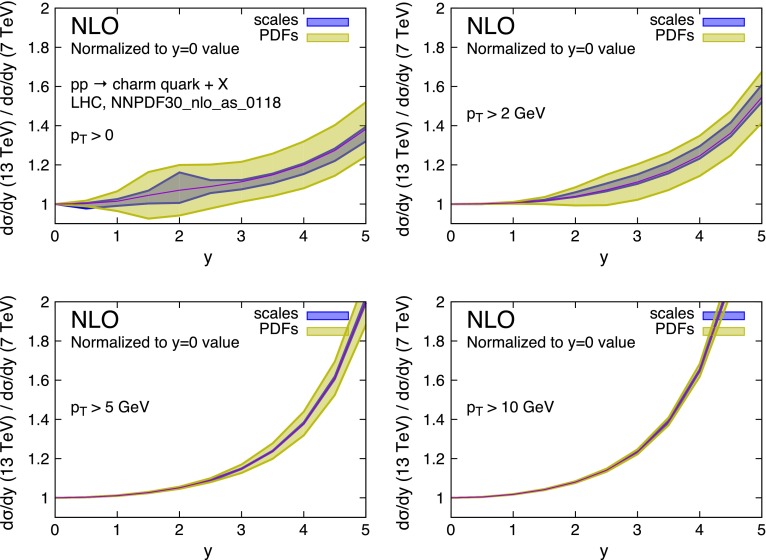
Same as Fig. 7, but with further normalisations to the values of the ratios at $y=0$

**Fig. 10:**
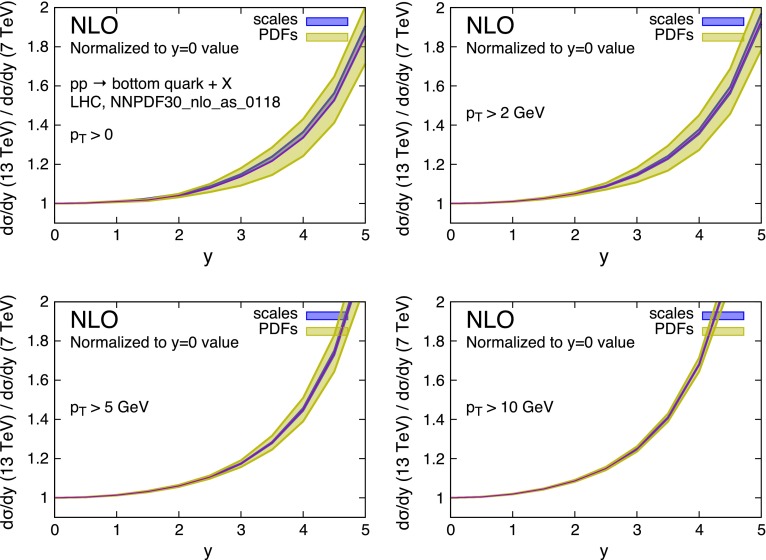
Same as Fig. 8, but with further normalisations to the values of the ratios at $y=0$

In order to reduce uncertainties even further, one can consider taking a double ratio, and normalise the 13-over-7 cross-section ratios to those measured at a given value of rapidity, as suggested in Ref. [31]. Figures 9 and 10 show that, when this is done (in this case using $y=0$ as a reference point) the PDF uncertainty remains by far the dominant one.

This suggests that the double ratio

3 $$\begin{aligned} \mathrm{RR}(y,\bar{y})=\frac{R(y)}{R(\bar{y})}, \end{aligned}$$

with $\bar{y}$ conveniently chosen, can represent a powerful handle for an even more precise determination of the gluon PDFs.

**Fig. 11:**
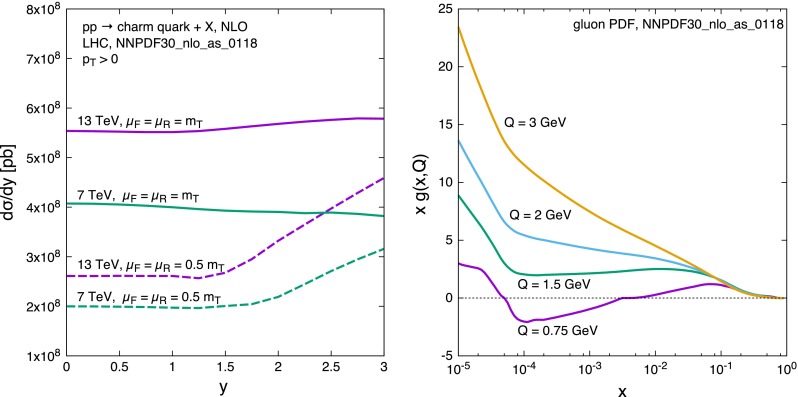
*Left plot* NLO $\mathrm{d}\sigma /\mathrm{d}y$ distributions for charm quark production in *pp* collisions at 7 and 13 TeV centre-of-mass energies. The curves predicted with a ‘central’ choice, $m_T$, for the factorisation and the renormalisation scales are shown (*solid lines*), as well as those given by the choice $\mu = m_T/2$ (*dashed lines*). *Right plot* the gluon parton distribution function of NNPDF30 at small scales

A feature that can be observed in Fig. 9 (and, to a lesser extent, also in Fig. 7) deserves an explanation. One can see how the scale variation band suddenly grows and then decreases around $y\simeq 2$. We have examined this behaviour in some detail, and found that it can be attributed to the behaviour of the gluon PDF at very small factorisation scale. Figure 11(left) shows the rapidity distribution of charm (integrated down to $p_T = 0$) at 7 and 13 TeV centre-of-mass energy and for two factorisation and renormalisation scale choices. For the low scale choice $\mu _F = \mu _R = m_T/2$ the rapidity distribution can be seen to start growing around $y\simeq 1.5$ and $y\simeq 2$ at 13 and 7 TeV respectively. This behaviour can be understood by looking at the gluon density displayed in Fig. 11(right): at small scales it displays a very different slope below and above $x\simeq 10^{-4}$. As the rapidity of the charm quark increases, the rapidity of the partonic system also increases, thus driving one of two momentum fractions $x_1$, $x_2$, say $x_1$, towards very small values, while $x_2$ grows. At the larger scale $Q=3\;$GeV this implies that $g(x_1)$ grows and $g(x_2)$ decreases, leading to a roughly constant luminosity, and thus to a constant rapidity distribution, as can be seen on the left plot. On the contrary, for smaller scales $g(x_2)$ is flat for $x_2 >10^{-4}$, and the luminosity grows proportionally to $g(x_1)$, leading to the cross section growth again shown in the left plot.

As the centre-of-mass energy grows, $x_1$ and $x_2$ are scaled to smaller values, so that the onset of the effect discussed in the previous paragraph is shifted in rapidity. This causes a mismatch in the cancellation of scale variation effects that takes place around $y\simeq 1.5$–2, leading to the feature shown in Fig. 9.

It is clear that this effect has to do with the behaviour of the parton densities at very small scales. On the other hand, we have observed the same behaviour also with the MMHT PDF set [45]. In this work we rely upon the correctness of the PDF’s in this region. However, further investigation of this issue in the framework of PDF fits may be desirable.

## FONLL transverse momentum distributions

The higher centre-of-mass energy of the Run 2 at the LHC will allow experiments to measure charm and bottom hadrons up to transverse momenta much larger than those observed during Run 1. Using an integrated luminosity of about 15 nb$^{-1}$ at $\sqrt{S}=7$ TeV, LHCb reported [26] precise spectrum measurements of *D* mesons up to cross sections $\mathrm{d}\sigma /\mathrm{d}y\mathrm{d}p_T$ of order 1$\mu$b/GeV. This suggests that integrated luminosities in the range of 1–2 fb$^{-1}$ should push the sensitivity up to rates $\mathrm{d}\sigma /\mathrm{d}y\mathrm{d}p_T \sim 10$ pb/GeV. This means $p_T$ values above 30 GeV, even for the largest rapidity ranges accessible to LHCb. At these $p_T$ values, much larger than the charm quark mass, resummation of multiple quasi-collinear gluon emission is necessary. We therefore provide FONLL [38] predictions for the $p_T$ distributions of charm mesons, and for ratios of $p_T$ distributions, to be compared with future data. The details of these calculations are documented in Ref. [28]. We present here the results in graphical form, and we collect them in the appendix in tabular form for easier use, integrated over finite bins of $p_T$ and *y*, and inclusive of scale, mass and PDF uncertainties.

**Fig. 12:**
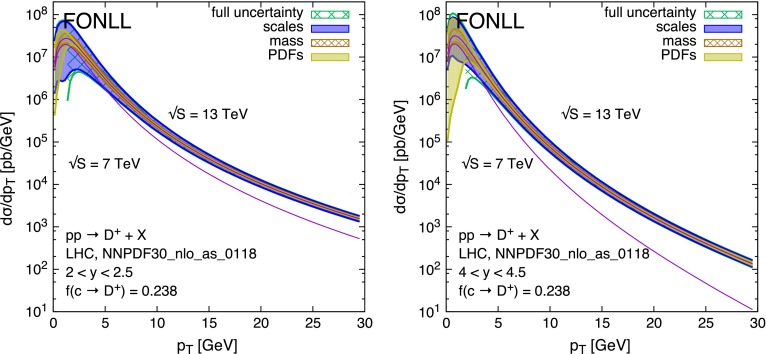
Transverse momentum distributions of $D^+$ mesons in *pp* collisions at $\sqrt{S}=13$ TeV collisions in the LHC, in the rapidity regions $2 < y < 2.5$ (*left plot*) and $4 < y < 4.5$ (*right plot*). The predictions at 7 TeV are also shown for comparison

Figure 12 shows the $p_T$ distribution of $D^+$ mesons at $\sqrt{S}=13$ and 7 TeV, in the rapidity ranges $2 < y < 2.5$ and $4 < y < 4.5$, i.e. the first and the last bin where LHCb reported results during Run 1.

**Fig. 13:**
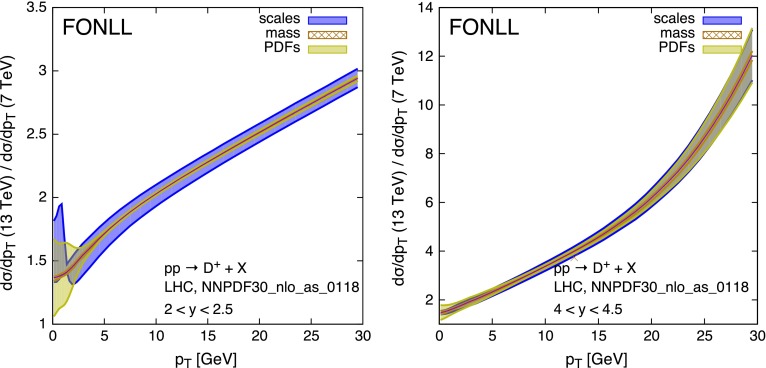
Ratios of transverse momentum distributions of $D^+$ mesons in *pp* collisions at $\sqrt{S}=13$ TeV and $\sqrt{S}=7$ TeV collisions in the LHC, in the rapidity regions $2 < y < 2.5$ (*left plot*) and $4 < y < 4.5$ (*right plot*)

Figure 13 shows the ratio of the same distributions at 13 and at 7 TeV, showing to what extent the uncertainties originating from scale variations and from PDFs do cancel. The interesting observation here is that, in the most forward rapidity bin, $4 < y < 4.5$, the uncertainty from PDFs is the dominant one at very small transverse momentum (i.e. $p_T\lesssim 5$ GeV), consistently with what we observed earlier, but it is also still commensurate with the scale uncertainty at large $p_T$ (i.e. $p_T\gtrsim 20$ GeV). As shown in Fig. 1, in this high-*y* and high-$p_T$ region one is in fact probing the gluon density in the less constrained domain of $x\gtrsim 0.2$.

Notice that, in order to fully exploit the sensitivity to PDFs via the cross-section ratios at high-$p_T$, it will be necessary to increase the statistics used for the 7 (or 8) TeV measurements from the $\mathcal{O}(10\,\, \mathrm {nb}^{-1})$ of the existing publications, to the $\mathcal{O}(1\,\, \mathrm {fb}^{-1})$ of the full available dataset. In the low-$p_T$ region, on the other hand, it may also be useful to exploit the double ratios RR$(y,\bar{y})$, as suggested in Sect. 3. A normalisation point at $\bar{y}=0$ can be provided by measurements from the ALICE experiment, which has sensitivity down to very low $p_T$ values.

## Conclusions

In spite of the well-known limited theoretical precision, we have given evidence in this paper that measurements of appropriate observables built upon open heavy-quark production rates offer a great potential for sensitive tests of and constraints on the gluon PDF. The cross-section ratios that we have introduced are very robust with respect to higher-order corrections, in spite of the large *K* factors that characterise the absolute rates at a given energy. The step in energy from $\sqrt{S}=7$ to 13 TeV is large enough that the rate comparisons at the same value of *y* and $p_T$ can expose small differences in the *x*-distributions of the members of current PDF sets. In several cases, these differences can affect the predictions for cross-section ratios at the 5–10 % level, larger than the other sources of theoretical systematics. While experimental measurements at this level of precision are challenging, we expect that suitable analyses of ratios should be doable, to benefit from similar cancellations of the systematics of experimental origin.

## Appendix

This appendix contains the values of the FONLL predictions for $D^+$ and $B^+$ production cross sections in *pp* collisions at 13 TeV, and the ratios with the corresponding predictions at 7 TeV. Cross sections and ratios for $D^0$ and $D^*$ are not included here for brevity, but they are qualitatively very similar and can be obtained from the authors.

**Table:**

FONLL $pp\rightarrow D^+ X$, $\sigma ~(13~\mathrm {TeV})$ [pb], $2 < y < 2.5$, $f(c\rightarrow D^+) = 0.238$										
$p_T^\mathrm{min}$	$p_T^\mathrm{max}$	$y^\mathrm{min}$	$y^\mathrm{max}$	Central	Scales$-$	Scales$+$	Mass$-$	Mass$+$	PDFs$-$	PDFs$+$
0.00	0.25	2.00	2.50	$1.717\times 10^6$	$3.418\times 10^5$	$5.914\times 10^6$	$1.463\times 10^6$	$1.865\times 10^6$	$1.047\times 10^5$	$4.115\times 10^6$
0.25	0.50	2.00	2.50	$4.034\times 10^6$	$6.671\times 10^5$	$1.270\times 10^7$	$3.339\times 10^6$	$4.608\times 10^6$	$3.668\times 10^5$	$8.908\times 10^6$
0.50	0.75	2.00	2.50	$5.522\times 10^6$	$7.057\times 10^5$	$1.587\times 10^7$	$4.453\times 10^6$	$6.631\times 10^6$	$8.597\times 10^5$	$1.081\times 10^7$
0.75	1.00	2.00	2.50	$6.466\times 10^6$	$6.700\times 10^5$	$1.735\times 10^7$	$5.081\times 10^6$	$8.154\times 10^6$	$1.953\times 10^6$	$1.100\times 10^7$
1.00	1.25	2.00	2.50	$6.916\times 10^6$	$7.147\times 10^5$	$1.758\times 10^7$	$5.343\times 10^6$	$9.011\times 10^6$	$3.499\times 10^6$	$1.035\times 10^7$
1.25	1.50	2.00	2.50	$6.869\times 10^6$	$8.839\times 10^5$	$1.663\times 10^7$	$5.276\times 10^6$	$9.092\times 10^6$	$4.482\times 10^6$	$9.258\times 10^6$
1.50	1.75	2.00	2.50	$6.447\times 10^6$	$1.075\times 10^6$	$1.492\times 10^7$	$4.967\times 10^6$	$8.563\times 10^6$	$4.850\times 10^6$	$8.040\times 10^6$
1.75	2.00	2.00	2.50	$5.810\times 10^6$	$1.207\times 10^6$	$1.289\times 10^7$	$4.505\times 10^6$	$7.683\times 10^6$	$4.772\times 10^6$	$6.852\times 10^6$
2.00	2.25	2.00	2.50	$5.091\times 10^6$	$1.271\times 10^6$	$1.086\times 10^7$	$3.984\times 10^6$	$6.678\times 10^6$	$4.410\times 10^6$	$5.774\times 10^6$
2.25	2.50	2.00	2.50	$4.379\times 10^6$	$1.284\times 10^6$	$9.013\times 10^6$	$3.461\times 10^6$	$5.705\times 10^6$	$3.927\times 10^6$	$4.834\times 10^6$
2.50	2.75	2.00	2.50	$3.725\times 10^6$	$1.240\times 10^6$	$7.421\times 10^6$	$2.973\times 10^6$	$4.793\times 10^6$	$3.415\times 10^6$	$4.036\times 10^6$
2.75	3.00	2.00	2.50	$3.149\times 10^6$	$1.155\times 10^6$	$6.088\times 10^6$	$2.535\times 10^6$	$4.001\times 10^6$	$2.932\times 10^6$	$3.365\times 10^6$
3.00	4.00	2.00	2.50	$8.316\times 10^6$	$3.637\times 10^6$	$1.520\times 10^7$	$6.816\times 10^6$	$1.041\times 10^7$	$7.894\times 10^6$	$8.742\times 10^6$
4.00	5.00	2.00	2.50	$4.205\times 10^6$	$2.280\times 10^6$	$7.095\times 10^6$	$3.544\times 10^6$	$5.119\times 10^6$	$4.053\times 10^6$	$4.358\times 10^6$
5.00	6.00	2.00	2.50	$2.235\times 10^6$	$1.388\times 10^6$	$3.549\times 10^6$	$1.927\times 10^6$	$2.656\times 10^6$	$2.167\times 10^6$	$2.302\times 10^6$
6.00	7.00	2.00	2.50	$1.254\times 10^6$	$8.537\times 10^5$	$1.900\times 10^6$	$1.101\times 10^6$	$1.461\times 10^6$	$1.220\times 10^6$	$1.288\times 10^6$
7.00	8.00	2.00	2.50	$7.385\times 10^5$	$5.360\times 10^5$	$1.078\times 10^6$	$6.583\times 10^5$	$8.475\times 10^5$	$7.192\times 10^5$	$7.578\times 10^5$
8.00	9.00	2.00	2.50	$4.541\times 10^5$	$3.399\times 10^5$	$6.424\times 10^5$	$4.096\times 10^5$	$5.143\times 10^5$	$4.424\times 10^5$	$4.655\times 10^5$
9.00	10.00	2.00	2.50	$2.899\times 10^5$	$2.208\times 10^5$	$3.994\times 10^5$	$2.642\times 10^5$	$3.249\times 10^5$	$2.825\times 10^5$	$2.970\times 10^5$
10.00	11.00	2.00	2.50	$1.911\times 10^5$	$1.477\times 10^5$	$2.575\times 10^5$	$1.756\times 10^5$	$2.125\times 10^5$	$1.863\times 10^5$	$1.959\times 10^5$
11.00	12.00	2.00	2.50	$1.297\times 10^5$	$1.015\times 10^5$	$1.715\times 10^5$	$1.200\times 10^5$	$1.432\times 10^5$	$1.264\times 10^5$	$1.329\times 10^5$
12.00	13.00	2.00	2.50	$9.020\times 10^4$	$7.140\times 10^4$	$1.174\times 10^5$	$8.397\times 10^4$	$9.901\times 10^4$	$8.789\times 10^4$	$9.249\times 10^4$
13.00	14.00	2.00	2.50	$6.414\times 10^4$	$5.127\times 10^4$	$8.228\times 10^4$	$6.002\times 10^4$	$7.009\times 10^4$	$6.250\times 10^4$	$6.578\times 10^4$
14.00	15.00	2.00	2.50	$4.653\times 10^4$	$3.751\times 10^4$	$5.893\times 10^4$	$4.372\times 10^4$	$5.065\times 10^4$	$4.534\times 10^4$	$4.774\times 10^4$
15.00	16.00	2.00	2.50	$3.434\times 10^4$	$2.789\times 10^4$	$4.301\times 10^4$	$3.239\times 10^4$	$3.727\times 10^4$	$3.346\times 10^4$	$3.525\times 10^4$
16.00	17.00	2.00	2.50	$2.575\times 10^4$	$2.107\times 10^4$	$3.194\times 10^4$	$2.435\times 10^4$	$2.792\times 10^4$	$2.509\times 10^4$	$2.644\times 10^4$
17.00	18.00	2.00	2.50	$1.959\times 10^4$	$1.612\times 10^4$	$2.409\times 10^4$	$1.857\times 10^4$	$2.121\times 10^4$	$1.907\times 10^4$	$2.011\times 10^4$
18.00	19.00	2.00	2.50	$1.509\times 10^4$	$1.249\times 10^4$	$1.840\times 10^4$	$1.434\times 10^4$	$1.634\times 10^4$	$1.469\times 10^4$	$1.549\times 10^4$
19.00	20.00	2.00	2.50	$1.176\times 10^4$	$9.787\times 10^3$	$1.424\times 10^4$	$1.120\times 10^4$	$1.274\times 10^4$	$1.145\times 10^4$	$1.208\times 10^4$
20.00	21.00	2.00	2.50	$9.265\times 10^3$	$7.745\times 10^3$	$1.114\times 10^4$	$8.835\times 10^3$	$1.003\times 10^4$	$9.015\times 10^3$	$9.515\times 10^3$
21.00	22.00	2.00	2.50	$7.371\times 10^3$	$6.186\times 10^3$	$8.811\times 10^3$	$7.038\times 10^3$	$7.968\times 10^3$	$7.171\times 10^3$	$7.571\times 10^3$
22.00	23.00	2.00	2.50	$5.917\times 10^3$	$4.984\times 10^3$	$7.033\times 10^3$	$5.655\times 10^3$	$6.378\times 10^3$	$5.755\times 10^3$	$6.076\times 10^3$
23.00	24.00	2.00	2.50	$4.786\times 10^3$	$4.048\times 10^3$	$5.662\times 10^3$	$4.582\times 10^3$	$5.143\times 10^3$	$4.655\times 10^3$	$4.917\times 10^3$
24.00	25.00	2.00	2.50	$3.903\times 10^3$	$3.311\times 10^3$	$4.596\times 10^3$	$3.739\times 10^3$	$4.179\times 10^3$	$3.796\times 10^3$	$4.010\times 10^3$
25.00	26.00	2.00	2.50	$3.203\times 10^3$	$2.727\times 10^3$	$3.758\times 10^3$	$3.073\times 10^3$	$3.422\times 10^3$	$3.115\times 10^3$	$3.292\times 10^3$
26.00	27.00	2.00	2.50	$2.649\times 10^3$	$2.260\times 10^3$	$3.094\times 10^3$	$2.542\times 10^3$	$2.823\times 10^3$	$2.575\times 10^3$	$2.720\times 10^3$
27.00	28.00	2.00	2.50	$2.202\times 10^3$	$1.884\times 10^3$	$2.563\times 10^3$	$2.115\times 10^3$	$2.343\times 10^3$	$2.142\times 10^3$	$2.263\times 10^3$
28.00	29.00	2.00	2.50	$1.842\times 10^3$	$1.580\times 10^3$	$2.136\times 10^3$	$1.770\times 10^3$	$1.958\times 10^3$	$1.791\times 10^3$	$1.893\times 10^3$
29.00	30.00	2.00	2.50	$1.548\times 10^3$	$1.331\times 10^3$	$1.790\times 10^3$	$1.489\times 10^3$	$1.646\times 10^3$	$1.505\times 10^3$	$1.591\times 10^3$

**Table:**

FONLL $pp\rightarrow D^+ X$, $\sigma ~(13~\mathrm {TeV})$ [pb], $2.5 < y < 3$, $f(c\rightarrow D^+) = 0.238$										
$p_T^\mathrm{min}$	$p_T^\mathrm{max}$	$y^\mathrm{min}$	$y^\mathrm{max}$	Central	Scales$-$	Scales$+$	Mass$-$	Mass$+$	PDFs$-$	PDFs$+$
0.00	0.25	2.50	3.00	$2.010\times 10^6$	$5.422\times 10^5$	$6.881\times 10^6$	$1.642\times 10^6$	$2.329\times 10^6$	$7.547\times 10^4$	$5.276\times 10^6$
0.25	0.50	2.50	3.00	$4.629\times 10^6$	$1.069\times 10^6$	$1.446\times 10^7$	$3.687\times 10^6$	$5.591\times 10^6$	$2.637\times 10^5$	$1.129\times 10^7$
0.50	0.75	2.50	3.00	$6.157\times 10^6$	$1.139\times 10^6$	$1.755\times 10^7$	$4.810\times 10^6$	$7.728\times 10^6$	$5.536\times 10^5$	$1.345\times 10^7$
0.75	1.00	2.50	3.00	$6.983\times 10^6$	$1.058\times 10^6$	$1.859\times 10^7$	$5.362\times 10^6$	$9.096\times 10^6$	$1.136\times 10^6$	$1.335\times 10^7$
1.00	1.25	2.50	3.00	$7.254\times 10^6$	$1.053\times 10^6$	$1.833\times 10^7$	$5.512\times 10^6$	$9.684\times 10^6$	$2.331\times 10^6$	$1.216\times 10^7$
1.25	1.50	2.50	3.00	$7.028\times 10^6$	$1.180\times 10^6$	$1.694\times 10^7$	$5.334\times 10^6$	$9.480\times 10^6$	$3.549\times 10^6$	$1.052\times 10^7$
1.50	1.75	2.50	3.00	$6.466\times 10^6$	$1.316\times 10^6$	$1.491\times 10^7$	$4.931\times 10^6$	$8.694\times 10^6$	$4.096\times 10^6$	$8.832\times 10^6$
1.75	2.00	2.50	3.00	$5.729\times 10^6$	$1.383\times 10^6$	$1.267\times 10^7$	$4.410\times 10^6$	$7.647\times 10^6$	$4.158\times 10^6$	$7.297\times 10^6$
2.00	2.25	2.50	3.00	$4.950\times 10^6$	$1.385\times 10^6$	$1.054\times 10^7$	$3.848\times 10^6$	$6.552\times 10^6$	$3.915\times 10^6$	$5.981\times 10^6$
2.25	2.50	2.50	3.00	$4.208\times 10^6$	$1.349\times 10^6$	$8.651\times 10^6$	$3.308\times 10^6$	$5.510\times 10^6$	$3.525\times 10^6$	$4.891\times 10^6$
2.50	2.75	2.50	3.00	$3.544\times 10^6$	$1.267\times 10^6$	$7.054\times 10^6$	$2.813\times 10^6$	$4.582\times 10^6$	$3.084\times 10^6$	$4.001\times 10^6$
2.75	3.00	2.50	3.00	$2.968\times 10^6$	$1.155\times 10^6$	$5.738\times 10^6$	$2.379\times 10^6$	$3.796\times 10^6$	$2.656\times 10^6$	$3.277\times 10^6$
3.00	4.00	2.50	3.00	$7.714\times 10^6$	$3.513\times 10^6$	$1.410\times 10^7$	$6.302\times 10^6$	$9.696\times 10^6$	$7.152\times 10^6$	$8.275\times 10^6$
4.00	5.00	2.50	3.00	$3.818\times 10^6$	$2.121\times 10^6$	$6.445\times 10^6$	$3.208\times 10^6$	$4.665\times 10^6$	$3.644\times 10^6$	$3.994\times 10^6$
5.00	6.00	2.50	3.00	$1.995\times 10^6$	$1.262\times 10^6$	$3.170\times 10^6$	$1.716\times 10^6$	$2.378\times 10^6$	$1.924\times 10^6$	$2.067\times 10^6$
6.00	7.00	2.50	3.00	$1.104\times 10^6$	$7.628\times 10^5$	$1.674\times 10^6$	$9.675\times 10^5$	$1.290\times 10^6$	$1.070\times 10^6$	$1.138\times 10^6$
7.00	8.00	2.50	3.00	$6.424\times 10^5$	$4.712\times 10^5$	$9.380\times 10^5$	$5.714\times 10^5$	$7.390\times 10^5$	$6.238\times 10^5$	$6.609\times 10^5$
8.00	9.00	2.50	3.00	$3.906\times 10^5$	$2.923\times 10^5$	$5.529\times 10^5$	$3.518\times 10^5$	$4.434\times 10^5$	$3.798\times 10^5$	$4.013\times 10^5$
9.00	10.00	2.50	3.00	$2.468\times 10^5$	$1.879\times 10^5$	$3.401\times 10^5$	$2.245\times 10^5$	$2.773\times 10^5$	$2.401\times 10^5$	$2.532\times 10^5$
10.00	11.00	2.50	3.00	$1.610\times 10^5$	$1.244\times 10^5$	$2.172\times 10^5$	$1.478\times 10^5$	$1.794\times 10^5$	$1.568\times 10^5$	$1.652\times 10^5$
11.00	12.00	2.50	3.00	$1.082\times 10^5$	$8.468\times 10^4$	$1.432\times 10^5$	$1.000\times 10^5$	$1.197\times 10^5$	$1.054\times 10^5$	$1.110\times 10^5$
12.00	13.00	2.50	3.00	$7.454\times 10^4$	$5.898\times 10^4$	$9.706\times 10^4$	$6.931\times 10^4$	$8.201\times 10^4$	$7.266\times 10^4$	$7.645\times 10^4$
13.00	14.00	2.50	3.00	$5.253\times 10^4$	$4.196\times 10^4$	$6.743\times 10^4$	$4.908\times 10^4$	$5.752\times 10^4$	$5.119\times 10^4$	$5.384\times 10^4$
14.00	15.00	2.50	3.00	$3.775\times 10^4$	$3.042\times 10^4$	$4.784\times 10^4$	$3.541\times 10^4$	$4.117\times 10^4$	$3.679\times 10^4$	$3.870\times 10^4$
15.00	16.00	2.50	3.00	$2.761\times 10^4$	$2.242\times 10^4$	$3.461\times 10^4$	$2.599\times 10^4$	$2.999\times 10^4$	$2.692\times 10^4$	$2.830\times 10^4$
16.00	17.00	2.50	3.00	$2.052\times 10^4$	$1.677\times 10^4$	$2.547\times 10^4$	$1.938\times 10^4$	$2.221\times 10^4$	$2.001\times 10^4$	$2.103\times 10^4$
17.00	18.00	2.50	3.00	$1.547\times 10^4$	$1.272\times 10^4$	$1.903\times 10^4$	$1.464\times 10^4$	$1.671\times 10^4$	$1.508\times 10^4$	$1.585\times 10^4$
18.00	19.00	2.50	3.00	$1.181\times 10^4$	$9.770\times 10^3$	$1.442\times 10^4$	$1.121\times 10^4$	$1.276\times 10^4$	$1.152\times 10^4$	$1.210\times 10^4$
19.00	20.00	2.50	3.00	$9.127\times 10^3$	$7.585\times 10^3$	$1.106\times 10^4$	$8.677\times 10^3$	$9.877\times 10^3$	$8.901\times 10^3$	$9.351\times 10^3$
20.00	21.00	2.50	3.00	$7.128\times 10^3$	$5.952\times 10^3$	$8.582\times 10^3$	$6.788\times 10^3$	$7.725\times 10^3$	$6.952\times 10^3$	$7.302\times 10^3$
21.00	22.00	2.50	3.00	$5.622\times 10^3$	$4.715\times 10^3$	$6.728\times 10^3$	$5.362\times 10^3$	$6.090\times 10^3$	$5.484\times 10^3$	$5.760\times 10^3$
22.00	23.00	2.50	3.00	$4.474\times 10^3$	$3.765\times 10^3$	$5.326\times 10^3$	$4.272\times 10^3$	$4.834\times 10^3$	$4.365\times 10^3$	$4.584\times 10^3$
23.00	24.00	2.50	3.00	$3.591\times 10^3$	$3.032\times 10^3$	$4.253\times 10^3$	$3.432\times 10^3$	$3.868\times 10^3$	$3.503\times 10^3$	$3.677\times 10^3$
24.00	25.00	2.50	3.00	$2.904\times 10^3$	$2.461\times 10^3$	$3.425\times 10^3$	$2.780\times 10^3$	$3.115\times 10^3$	$2.832\times 10^3$	$2.975\times 10^3$
25.00	26.00	2.50	3.00	$2.365\times 10^3$	$2.010\times 10^3$	$2.777\times 10^3$	$2.265\times 10^3$	$2.530\times 10^3$	$2.306\times 10^3$	$2.423\times 10^3$
26.00	27.00	2.50	3.00	$1.938\times 10^3$	$1.653\times 10^3$	$2.268\times 10^3$	$1.859\times 10^3$	$2.069\times 10^3$	$1.891\times 10^3$	$1.986\times 10^3$
27.00	28.00	2.50	3.00	$1.599\times 10^3$	$1.367\times 10^3$	$1.864\times 10^3$	$1.534\times 10^3$	$1.705\times 10^3$	$1.560\times 10^3$	$1.638\times 10^3$
28.00	29.00	2.50	3.00	$1.327\times 10^3$	$1.137\times 10^3$	$1.542\times 10^3$	$1.274\times 10^3$	$1.414\times 10^3$	$1.294\times 10^3$	$1.359\times 10^3$
29.00	30.00	2.50	3.00	$1.107\times 10^3$	$9.508\times 10^2$	$1.282\times 10^3$	$1.063\times 10^3$	$1.179\times 10^3$	$1.079\times 10^3$	$1.134\times 10^3$

**Table:**

FONLL $pp\rightarrow D^+ X$, $\sigma ~(13~\mathrm {TeV})$ [pb], $3 < y < 3.5$, $f(c\rightarrow D^+) = 0.238$										
$p_T^\mathrm{min}$	$p_T^\mathrm{max}$	$y^\mathrm{min}$	$y^\mathrm{max}$	Central	Scales$-$	Scales$+$	Mass$-$	Mass$+$	PDFs$-$	PDFs$+$
0.00	0.25	3.00	3.50	$2.315\times 10^6$	$7.687\times 10^5$	$7.868\times 10^6$	$1.815\times 10^6$	$2.844\times 10^6$	$5.524\times 10^4$	$6.447\times 10^6$
0.25	0.50	3.00	3.50	$5.231\times 10^6$	$1.522\times 10^6$	$1.624\times 10^7$	$4.015\times 10^6$	$6.669\times 10^6$	$1.889\times 10^5$	$1.368\times 10^7$
0.50	0.75	3.00	3.50	$6.773\times 10^6$	$1.621\times 10^6$	$1.919\times 10^7$	$5.131\times 10^6$	$8.880\times 10^6$	$3.865\times 10^5$	$1.605\times 10^7$
0.75	1.00	3.00	3.50	$7.449\times 10^6$	$1.477\times 10^6$	$1.972\times 10^7$	$5.586\times 10^6$	$1.003\times 10^7$	$7.226\times 10^5$	$1.559\times 10^7$
1.00	1.25	3.00	3.50	$7.514\times 10^6$	$1.399\times 10^6$	$1.888\times 10^7$	$5.605\times 10^6$	$1.028\times 10^7$	$1.438\times 10^6$	$1.384\times 10^7$
1.25	1.50	3.00	3.50	$7.095\times 10^6$	$1.466\times 10^6$	$1.702\times 10^7$	$5.310\times 10^6$	$9.727\times 10^6$	$2.551\times 10^6$	$1.164\times 10^7$
1.50	1.75	3.00	3.50	$6.383\times 10^6$	$1.535\times 10^6$	$1.467\times 10^7$	$4.817\times 10^6$	$8.699\times 10^6$	$3.268\times 10^6$	$9.503\times 10^6$
1.75	2.00	3.00	3.50	$5.553\times 10^6$	$1.530\times 10^6$	$1.226\times 10^7$	$4.236\times 10^6$	$7.495\times 10^6$	$3.458\times 10^6$	$7.645\times 10^6$
2.00	2.25	3.00	3.50	$4.722\times 10^6$	$1.470\times 10^6$	$1.004\times 10^7$	$3.646\times 10^6$	$6.300\times 10^6$	$3.330\times 10^6$	$6.117\times 10^6$
2.25	2.50	3.00	3.50	$3.960\times 10^6$	$1.384\times 10^6$	$8.137\times 10^6$	$3.094\times 10^6$	$5.207\times 10^6$	$3.032\times 10^6$	$4.891\times 10^6$
2.50	2.75	3.00	3.50	$3.296\times 10^6$	$1.266\times 10^6$	$6.562\times 10^6$	$2.604\times 10^6$	$4.284\times 10^6$	$2.670\times 10^6$	$3.925\times 10^6$
2.75	3.00	3.00	3.50	$2.732\times 10^6$	$1.131\times 10^6$	$5.284\times 10^6$	$2.181\times 10^6$	$3.518\times 10^6$	$2.306\times 10^6$	$3.161\times 10^6$
3.00	4.00	3.00	3.50	$6.971\times 10^6$	$3.322\times 10^6$	$1.275\times 10^7$	$5.669\times 10^6$	$8.808\times 10^6$	$6.214\times 10^6$	$7.730\times 10^6$
4.00	5.00	3.00	3.50	$3.356\times 10^6$	$1.922\times 10^6$	$5.672\times 10^6$	$2.811\times 10^6$	$4.120\times 10^6$	$3.139\times 10^6$	$3.575\times 10^6$
5.00	6.00	3.00	3.50	$1.716\times 10^6$	$1.109\times 10^6$	$2.730\times 10^6$	$1.471\times 10^6$	$2.053\times 10^6$	$1.637\times 10^6$	$1.795\times 10^6$
6.00	7.00	3.00	3.50	$9.311\times 10^5$	$6.550\times 10^5$	$1.413\times 10^6$	$8.135\times 10^5$	$1.092\times 10^6$	$8.965\times 10^5$	$9.658\times 10^5$
7.00	8.00	3.00	3.50	$5.324\times 10^5$	$3.903\times 10^5$	$7.785\times 10^5$	$4.724\times 10^5$	$6.148\times 10^5$	$5.150\times 10^5$	$5.498\times 10^5$
8.00	9.00	3.00	3.50	$3.184\times 10^5$	$2.382\times 10^5$	$4.515\times 10^5$	$2.861\times 10^5$	$3.630\times 10^5$	$3.089\times 10^5$	$3.280\times 10^5$
9.00	10.00	3.00	3.50	$1.980\times 10^5$	$1.507\times 10^5$	$2.735\times 10^5$	$1.798\times 10^5$	$2.233\times 10^5$	$1.925\times 10^5$	$2.036\times 10^5$
10.00	11.00	3.00	3.50	$1.273\times 10^5$	$9.827\times 10^4$	$1.720\times 10^5$	$1.166\times 10^5$	$1.423\times 10^5$	$1.239\times 10^5$	$1.307\times 10^5$
11.00	12.00	3.00	3.50	$8.428\times 10^4$	$6.588\times 10^4$	$1.118\times 10^5$	$7.773\times 10^4$	$9.356\times 10^4$	$8.209\times 10^4$	$8.647\times 10^4$
12.00	13.00	3.00	3.50	$5.724\times 10^4$	$4.522\times 10^4$	$7.468\times 10^4$	$5.310\times 10^4$	$6.319\times 10^4$	$5.579\times 10^4$	$5.869\times 10^4$
13.00	14.00	3.00	3.50	$3.977\times 10^4$	$3.173\times 10^4$	$5.117\times 10^4$	$3.708\times 10^4$	$4.370\times 10^4$	$3.879\times 10^4$	$4.075\times 10^4$
14.00	15.00	3.00	3.50	$2.818\times 10^4$	$2.268\times 10^4$	$3.582\times 10^4$	$2.639\times 10^4$	$3.084\times 10^4$	$2.751\times 10^4$	$2.887\times 10^4$
15.00	16.00	3.00	3.50	$2.034\times 10^4$	$1.649\times 10^4$	$2.556\times 10^4$	$1.912\times 10^4$	$2.217\times 10^4$	$1.985\times 10^4$	$2.083\times 10^4$
16.00	17.00	3.00	3.50	$1.492\times 10^4$	$1.217\times 10^4$	$1.857\times 10^4$	$1.406\times 10^4$	$1.621\times 10^4$	$1.456\times 10^4$	$1.527\times 10^4$
17.00	18.00	3.00	3.50	$1.110\times 10^4$	$9.111\times 10^3$	$1.369\times 10^4$	$1.049\times 10^4$	$1.204\times 10^4$	$1.084\times 10^4$	$1.136\times 10^4$
18.00	19.00	3.00	3.50	$8.366\times 10^3$	$6.904\times 10^3$	$1.024\times 10^4$	$7.923\times 10^3$	$9.077\times 10^3$	$8.168\times 10^3$	$8.561\times 10^3$
19.00	20.00	3.00	3.50	$6.381\times 10^3$	$5.296\times 10^3$	$7.756\times 10^3$	$6.055\times 10^3$	$6.933\times 10^3$	$6.231\times 10^3$	$6.531\times 10^3$
20.00	21.00	3.00	3.50	$4.922\times 10^3$	$4.101\times 10^3$	$5.943\times 10^3$	$4.679\times 10^3$	$5.353\times 10^3$	$4.805\times 10^3$	$5.036\times 10^3$
21.00	22.00	3.00	3.50	$3.832\times 10^3$	$3.208\times 10^3$	$4.603\times 10^3$	$3.649\times 10^3$	$4.165\times 10^3$	$3.741\times 10^3$	$3.922\times 10^3$
22.00	23.00	3.00	3.50	$3.013\times 10^3$	$2.530\times 10^3$	$3.599\times 10^3$	$2.873\times 10^3$	$3.268\times 10^3$	$2.942\times 10^3$	$3.082\times 10^3$
23.00	24.00	3.00	3.50	$2.387\times 10^3$	$2.013\times 10^3$	$2.839\times 10^3$	$2.279\times 10^3$	$2.582\times 10^3$	$2.331\times 10^3$	$2.444\times 10^3$
24.00	25.00	3.00	3.50	$1.908\times 10^3$	$1.613\times 10^3$	$2.258\times 10^3$	$1.823\times 10^3$	$2.058\times 10^3$	$1.862\times 10^3$	$1.954\times 10^3$
25.00	26.00	3.00	3.50	$1.535\times 10^3$	$1.302\times 10^3$	$1.809\times 10^3$	$1.468\times 10^3$	$1.652\times 10^3$	$1.498\times 10^3$	$1.572\times 10^3$
26.00	27.00	3.00	3.50	$1.244\times 10^3$	$1.058\times 10^3$	$1.460\times 10^3$	$1.190\times 10^3$	$1.336\times 10^3$	$1.213\times 10^3$	$1.274\times 10^3$
27.00	28.00	3.00	3.50	$1.014\times 10^3$	$8.647\times 10^2$	$1.186\times 10^3$	$9.713\times 10^2$	$1.088\times 10^3$	$9.882\times 10^2$	$1.039\times 10^3$
28.00	29.00	3.00	3.50	$8.311\times 10^2$	$7.109\times 10^2$	$9.691\times 10^2$	$7.968\times 10^2$	$8.911\times 10^2$	$8.099\times 10^2$	$8.525\times 10^2$
29.00	30.00	3.00	3.50	$6.854\times 10^2$	$5.874\times 10^2$	$7.966\times 10^2$	$6.574\times 10^2$	$7.340\times 10^2$	$6.674\times 10^2$	$7.033\times 10^2$

**Table:**

FONLL $pp\rightarrow D^+ X$, $\sigma ~(13~\mathrm {TeV})$ [pb], $3.5 < y < 4$, $f(c\rightarrow D^+) = 0.238$										
$p_T^\mathrm{min}$	$p_T^\mathrm{max}$	$y^\mathrm{min}$	$y^\mathrm{max}$	Central	Scales$-$	Scales$+$	Mass$-$	Mass$+$	PDFs$-$	PDFs$+$
0.00	0.25	3.50	4.00	$2.620\times 10^6$	$1.023\times 10^6$	$8.851\times 10^6$	$1.968\times 10^6$	$3.411\times 10^6$	$3.891\times 10^4$	$7.602\times 10^6$
0.25	0.50	3.50	4.00	$5.810\times 10^6$	$2.027\times 10^6$	$1.796\times 10^7$	$4.289\times 10^6$	$7.816\times 10^6$	$1.314\times 10^5$	$1.599\times 10^7$
0.50	0.75	3.50	4.00	$7.326\times 10^6$	$2.151\times 10^6$	$2.067\times 10^7$	$5.372\times 10^6$	$1.005\times 10^7$	$2.616\times 10^5$	$1.847\times 10^7$
0.75	1.00	3.50	4.00	$7.804\times 10^6$	$1.922\times 10^6$	$2.056\times 10^7$	$5.705\times 10^6$	$1.090\times 10^7$	$4.662\times 10^5$	$1.754\times 10^7$
1.00	1.25	3.50	4.00	$7.630\times 10^6$	$1.747\times 10^6$	$1.909\times 10^7$	$5.583\times 10^6$	$1.072\times 10^7$	$8.606\times 10^5$	$1.518\times 10^7$
1.25	1.50	3.50	4.00	$7.009\times 10^6$	$1.730\times 10^6$	$1.676\times 10^7$	$5.167\times 10^6$	$9.794\times 10^6$	$1.628\times 10^6$	$1.244\times 10^7$
1.50	1.75	3.50	4.00	$6.159\times 10^6$	$1.716\times 10^6$	$1.412\times 10^7$	$4.593\times 10^6$	$8.518\times 10^6$	$2.420\times 10^6$	$9.898\times 10^6$
1.75	2.00	3.50	4.00	$5.250\times 10^6$	$1.634\times 10^6$	$1.157\times 10^7$	$3.967\times 10^6$	$7.180\times 10^6$	$2.730\times 10^6$	$7.768\times 10^6$
2.00	2.25	3.50	4.00	$4.389\times 10^6$	$1.511\times 10^6$	$9.318\times 10^6$	$3.361\times 10^6$	$5.902\times 10^6$	$2.701\times 10^6$	$6.074\times 10^6$
2.25	2.50	3.50	4.00	$3.625\times 10^6$	$1.377\times 10^6$	$7.440\times 10^6$	$2.811\times 10^6$	$4.803\times 10^6$	$2.494\times 10^6$	$4.755\times 10^6$
2.50	2.75	3.50	4.00	$2.977\times 10^6$	$1.228\times 10^6$	$5.921\times 10^6$	$2.336\times 10^6$	$3.901\times 10^6$	$2.212\times 10^6$	$3.741\times 10^6$
2.75	3.00	3.50	4.00	$2.437\times 10^6$	$1.074\times 10^6$	$4.712\times 10^6$	$1.933\times 10^6$	$3.161\times 10^6$	$1.915\times 10^6$	$2.961\times 10^6$
3.00	4.00	3.50	4.00	$6.074\times 10^6$	$3.034\times 10^6$	$1.111\times 10^7$	$4.912\times 10^6$	$7.725\times 10^6$	$5.148\times 10^6$	$7.000\times 10^6$
4.00	5.00	3.50	4.00	$2.825\times 10^6$	$1.671\times 10^6$	$4.779\times 10^6$	$2.355\times 10^6$	$3.487\times 10^6$	$2.568\times 10^6$	$3.082\times 10^6$
5.00	6.00	3.50	4.00	$1.402\times 10^6$	$9.301\times 10^5$	$2.234\times 10^6$	$1.197\times 10^6$	$1.687\times 10^6$	$1.315\times 10^6$	$1.489\times 10^6$
6.00	7.00	3.50	4.00	$7.409\times 10^5$	$5.293\times 10^5$	$1.127\times 10^6$	$6.447\times 10^5$	$8.737\times 10^5$	$7.057\times 10^5$	$7.759\times 10^5$
7.00	8.00	3.50	4.00	$4.132\times 10^5$	$3.023\times 10^5$	$6.057\times 10^5$	$3.651\times 10^5$	$4.793\times 10^5$	$3.970\times 10^5$	$4.291\times 10^5$
8.00	9.00	3.50	4.00	$2.413\times 10^5$	$1.801\times 10^5$	$3.432\times 10^5$	$2.160\times 10^5$	$2.763\times 10^5$	$2.331\times 10^5$	$2.494\times 10^5$
9.00	10.00	3.50	4.00	$1.467\times 10^5$	$1.113\times 10^5$	$2.033\times 10^5$	$1.327\times 10^5$	$1.662\times 10^5$	$1.422\times 10^5$	$1.512\times 10^5$
10.00	11.00	3.50	4.00	$9.225\times 10^4$	$7.102\times 10^4$	$1.252\times 10^5$	$8.418\times 10^4$	$1.036\times 10^5$	$8.961\times 10^4$	$9.489\times 10^4$
11.00	12.00	3.50	4.00	$5.979\times 10^4$	$4.658\times 10^4$	$7.963\times 10^4$	$5.495\times 10^4$	$6.666\times 10^4$	$5.814\times 10^4$	$6.140\times 10^4$
12.00	13.00	3.50	4.00	$3.975\times 10^4$	$3.130\times 10^4$	$5.210\times 10^4$	$3.677\times 10^4$	$4.405\times 10^4$	$3.870\times 10^4$	$4.079\times 10^4$
13.00	14.00	3.50	4.00	$2.706\times 10^4$	$2.151\times 10^4$	$3.496\times 10^4$	$2.516\times 10^4$	$2.985\times 10^4$	$2.635\times 10^4$	$2.775\times 10^4$
14.00	15.00	3.50	4.00	$1.878\times 10^4$	$1.506\times 10^4$	$2.399\times 10^4$	$1.754\times 10^4$	$2.064\times 10^4$	$1.830\times 10^4$	$1.926\times 10^4$
15.00	16.00	3.50	4.00	$1.328\times 10^4$	$1.073\times 10^4$	$1.679\times 10^4$	$1.245\times 10^4$	$1.454\times 10^4$	$1.294\times 10^4$	$1.362\times 10^4$
16.00	17.00	3.50	4.00	$9.549\times 10^3$	$7.764\times 10^3$	$1.195\times 10^4$	$8.977\times 10^3$	$1.042\times 10^4$	$9.303\times 10^3$	$9.794\times 10^3$
17.00	18.00	3.50	4.00	$6.969\times 10^3$	$5.700\times 10^3$	$8.649\times 10^3$	$6.566\times 10^3$	$7.587\times 10^3$	$6.788\times 10^3$	$7.150\times 10^3$
18.00	19.00	3.50	4.00	$5.153\times 10^3$	$4.236\times 10^3$	$6.345\times 10^3$	$4.867\times 10^3$	$5.607\times 10^3$	$5.017\times 10^3$	$5.291\times 10^3$
19.00	20.00	3.50	4.00	$3.858\times 10^3$	$3.187\times 10^3$	$4.717\times 10^3$	$3.651\times 10^3$	$4.198\times 10^3$	$3.753\times 10^3$	$3.963\times 10^3$
20.00	21.00	3.50	4.00	$2.920\times 10^3$	$2.423\times 10^3$	$3.549\times 10^3$	$2.768\times 10^3$	$3.180\times 10^3$	$2.839\times 10^3$	$3.001\times 10^3$
21.00	22.00	3.50	4.00	$2.233\times 10^3$	$1.861\times 10^3$	$2.699\times 10^3$	$2.120\times 10^3$	$2.432\times 10^3$	$2.169\times 10^3$	$2.298\times 10^3$
22.00	23.00	3.50	4.00	$1.724\times 10^3$	$1.442\times 10^3$	$2.073\times 10^3$	$1.639\times 10^3$	$1.879\times 10^3$	$1.672\times 10^3$	$1.775\times 10^3$
23.00	24.00	3.50	4.00	$1.342\times 10^3$	$1.127\times 10^3$	$1.607\times 10^3$	$1.278\times 10^3$	$1.464\times 10^3$	$1.300\times 10^3$	$1.384\times 10^3$
24.00	25.00	3.50	4.00	$1.054\times 10^3$	$8.880\times 10^2$	$1.256\times 10^3$	$1.004\times 10^3$	$1.150\times 10^3$	$1.019\times 10^3$	$1.088\times 10^3$
25.00	26.00	3.50	4.00	$8.340\times 10^2$	$7.047\times 10^2$	$9.896\times 10^2$	$7.954\times 10^2$	$9.106\times 10^2$	$8.054\times 10^2$	$8.625\times 10^2$
26.00	27.00	3.50	4.00	$6.647\times 10^2$	$5.631\times 10^2$	$7.854\times 10^2$	$6.345\times 10^2$	$7.259\times 10^2$	$6.407\times 10^2$	$6.888\times 10^2$
27.00	28.00	3.50	4.00	$5.331\times 10^2$	$4.527\times 10^2$	$6.276\times 10^2$	$5.091\times 10^2$	$5.814\times 10^2$	$5.129\times 10^2$	$5.534\times 10^2$
28.00	29.00	3.50	4.00	$4.301\times 10^2$	$3.660\times 10^2$	$5.046\times 10^2$	$4.110\times 10^2$	$4.679\times 10^2$	$4.129\times 10^2$	$4.472\times 10^2$
29.00	30.00	3.50	4.00	$3.489\times 10^2$	$2.977\times 10^2$	$4.082\times 10^2$	$3.337\times 10^2$	$3.784\times 10^2$	$3.344\times 10^2$	$3.637\times 10^2$

**Table:**

FONLL $pp\rightarrow D^+ X$, $\sigma ~(13~\mathrm {TeV})$ [pb], $4 < y < 4.5$, $f(c\rightarrow D^+) = 0.238$										
$p_T^\mathrm{min}$	$p_T^\mathrm{max}$	$y^\mathrm{min}$	$y^\mathrm{max}$	Central	Scales$-$	Scales$+$	Mass$-$	Mass$+$	PDFs$-$	PDFs$+$
0.00	0.25	4.00	4.50	$2.885\times 10^6$	$1.291\times 10^6$	$9.729\times 10^6$	$2.076\times 10^6$	$3.986\times 10^6$	$2.637\times 10^4$	$8.651\times 10^6$
0.25	0.50	4.00	4.50	$6.290\times 10^6$	$2.551\times 10^6$	$1.941\times 10^7$	$4.462\times 10^6$	$8.944\times 10^6$	$8.820\times 10^4$	$1.800\times 10^7$
0.50	0.75	4.00	4.50	$7.721\times 10^6$	$2.692\times 10^6$	$2.175\times 10^7$	$5.474\times 10^6$	$1.111\times 10^7$	$1.722\times 10^5$	$2.045\times 10^7$
0.75	1.00	4.00	4.50	$7.963\times 10^6$	$2.360\times 10^6$	$2.092\times 10^7$	$5.664\times 10^6$	$1.153\times 10^7$	$2.987\times 10^5$	$1.898\times 10^7$
1.00	1.25	4.00	4.50	$7.537\times 10^6$	$2.068\times 10^6$	$1.880\times 10^7$	$5.403\times 10^6$	$1.086\times 10^7$	$5.096\times 10^5$	$1.601\times 10^7$
1.25	1.50	4.00	4.50	$6.724\times 10^6$	$1.944\times 10^6$	$1.603\times 10^7$	$4.874\times 10^6$	$9.589\times 10^6$	$9.482\times 10^5$	$1.277\times 10^7$
1.50	1.75	4.00	4.50	$5.757\times 10^6$	$1.835\times 10^6$	$1.317\times 10^7$	$4.234\times 10^6$	$8.099\times 10^6$	$1.616\times 10^6$	$9.898\times 10^6$
1.75	2.00	4.00	4.50	$4.796\times 10^6$	$1.672\times 10^6$	$1.055\times 10^7$	$3.582\times 10^6$	$6.638\times 10^6$	$2.015\times 10^6$	$7.578\times 10^6$
2.00	2.25	4.00	4.50	$3.929\times 10^6$	$1.490\times 10^6$	$8.335\times 10^6$	$2.980\times 10^6$	$5.343\times 10^6$	$2.073\times 10^6$	$5.786\times 10^6$
2.25	2.50	4.00	4.50	$3.189\times 10^6$	$1.316\times 10^6$	$6.543\times 10^6$	$2.451\times 10^6$	$4.272\times 10^6$	$1.947\times 10^6$	$4.432\times 10^6$
2.50	2.75	4.00	4.50	$2.578\times 10^6$	$1.142\times 10^6$	$5.127\times 10^6$	$2.006\times 10^6$	$3.411\times 10^6$	$1.739\times 10^6$	$3.415\times 10^6$
2.75	3.00	4.00	4.50	$2.081\times 10^6$	$9.770\times 10^5$	$4.022\times 10^6$	$1.637\times 10^6$	$2.723\times 10^6$	$1.508\times 10^6$	$2.651\times 10^6$
3.00	4.00	4.00	4.50	$5.034\times 10^6$	$2.644\times 10^6$	$9.227\times 10^6$	$4.044\times 10^6$	$6.457\times 10^6$	$4.027\times 10^6$	$6.040\times 10^6$
4.00	5.00	4.00	4.50	$2.237\times 10^6$	$1.372\times 10^6$	$3.791\times 10^6$	$1.854\times 10^6$	$2.782\times 10^6$	$1.964\times 10^6$	$2.511\times 10^6$
5.00	6.00	4.00	4.50	$1.066\times 10^6$	$7.273\times 10^5$	$1.705\times 10^6$	$9.054\times 10^5$	$1.293\times 10^6$	$9.772\times 10^5$	$1.155\times 10^6$
6.00	7.00	4.00	4.50	$5.429\times 10^5$	$3.868\times 10^5$	$8.297\times 10^5$	$4.698\times 10^5$	$6.447\times 10^5$	$5.091\times 10^5$	$5.767\times 10^5$
7.00	8.00	4.00	4.50	$2.923\times 10^5$	$2.131\times 10^5$	$4.308\times 10^5$	$2.570\times 10^5$	$3.415\times 10^5$	$2.777\times 10^5$	$3.068\times 10^5$
8.00	9.00	4.00	4.50	$1.650\times 10^5$	$1.226\times 10^5$	$2.360\times 10^5$	$1.469\times 10^5$	$1.902\times 10^5$	$1.581\times 10^5$	$1.719\times 10^5$
9.00	10.00	4.00	4.50	$9.698\times 10^4$	$7.328\times 10^4$	$1.354\times 10^5$	$8.732\times 10^4$	$1.106\times 10^5$	$9.339\times 10^4$	$1.006\times 10^5$
10.00	11.00	4.00	4.50	$5.905\times 10^4$	$4.522\times 10^4$	$8.071\times 10^4$	$5.362\times 10^4$	$6.676\times 10^4$	$5.702\times 10^4$	$6.107\times 10^4$
11.00	12.00	4.00	4.50	$3.708\times 10^4$	$2.873\times 10^4$	$4.979\times 10^4$	$3.392\times 10^4$	$4.160\times 10^4$	$3.587\times 10^4$	$3.827\times 10^4$
12.00	13.00	4.00	4.50	$2.390\times 10^4$	$1.872\times 10^4$	$3.161\times 10^4$	$2.201\times 10^4$	$2.668\times 10^4$	$2.313\times 10^4$	$2.468\times 10^4$
13.00	14.00	4.00	4.50	$1.579\times 10^4$	$1.248\times 10^4$	$2.061\times 10^4$	$1.462\times 10^4$	$1.753\times 10^4$	$1.528\times 10^4$	$1.630\times 10^4$
14.00	15.00	4.00	4.50	$1.065\times 10^4$	$8.492\times 10^3$	$1.374\times 10^4$	$9.903\times 10^3$	$1.178\times 10^4$	$1.030\times 10^4$	$1.100\times 10^4$
15.00	16.00	4.00	4.50	$7.326\times 10^3$	$5.881\times 10^3$	$9.349\times 10^3$	$6.838\times 10^3$	$8.075\times 10^3$	$7.078\times 10^3$	$7.573\times 10^3$
16.00	17.00	4.00	4.50	$5.127\times 10^3$	$4.144\times 10^3$	$6.478\times 10^3$	$4.798\times 10^3$	$5.633\times 10^3$	$4.946\times 10^3$	$5.305\times 10^3$
17.00	18.00	4.00	4.50	$3.641\times 10^3$	$2.961\times 10^3$	$4.565\times 10^3$	$3.418\times 10^3$	$3.994\times 10^3$	$3.508\times 10^3$	$3.777\times 10^3$
18.00	19.00	4.00	4.50	$2.625\times 10^3$	$2.144\times 10^3$	$3.265\times 10^3$	$2.468\times 10^3$	$2.870\times 10^3$	$2.520\times 10^3$	$2.727\times 10^3$
19.00	20.00	4.00	4.50	$1.914\times 10^3$	$1.572\times 10^3$	$2.366\times 10^3$	$1.805\times 10^3$	$2.089\times 10^3$	$1.835\times 10^3$	$1.994\times 10^3$
20.00	21.00	4.00	4.50	$1.413\times 10^3$	$1.165\times 10^3$	$1.736\times 10^3$	$1.334\times 10^3$	$1.538\times 10^3$	$1.351\times 10^3$	$1.476\times 10^3$
21.00	22.00	4.00	4.50	$1.055\times 10^3$	$8.735\times 10^2$	$1.288\times 10^3$	$9.977\times 10^2$	$1.146\times 10^3$	$1.005\times 10^3$	$1.105\times 10^3$
22.00	23.00	4.00	4.50	$7.947\times 10^2$	$6.607\times 10^2$	$9.656\times 10^2$	$7.528\times 10^2$	$8.625\times 10^2$	$7.542\times 10^2$	$8.351\times 10^2$
23.00	24.00	4.00	4.50	$6.036\times 10^2$	$5.038\times 10^2$	$7.302\times 10^2$	$5.726\times 10^2$	$6.552\times 10^2$	$5.705\times 10^2$	$6.369\times 10^2$
24.00	25.00	4.00	4.50	$4.622\times 10^2$	$3.870\times 10^2$	$5.567\times 10^2$	$4.389\times 10^2$	$5.024\times 10^2$	$4.348\times 10^2$	$4.896\times 10^2$
25.00	26.00	4.00	4.50	$3.568\times 10^2$	$2.994\times 10^2$	$4.279\times 10^2$	$3.391\times 10^2$	$3.887\times 10^2$	$3.339\times 10^2$	$3.798\times 10^2$
26.00	27.00	4.00	4.50	$2.775\times 10^2$	$2.334\times 10^2$	$3.318\times 10^2$	$2.639\times 10^2$	$3.030\times 10^2$	$2.582\times 10^2$	$2.970\times 10^2$
27.00	28.00	4.00	4.50	$2.172\times 10^2$	$1.829\times 10^2$	$2.587\times 10^2$	$2.066\times 10^2$	$2.375\times 10^2$	$2.008\times 10^2$	$2.337\times 10^2$
28.00	29.00	4.00	4.50	$1.708\times 10^2$	$1.441\times 10^2$	$2.029\times 10^2$	$1.625\times 10^2$	$1.869\times 10^2$	$1.567\times 10^2$	$1.849\times 10^2$
29.00	30.00	4.00	4.50	$1.349\times 10^2$	$1.141\times 10^2$	$1.599\times 10^2$	$1.285\times 10^2$	$1.476\times 10^2$	$1.228\times 10^2$	$1.471\times 10^2$

**Table:**

FONLL $pp\rightarrow D^+ + X$, $\sigma ~(13~\mathrm {TeV})/\sigma ~(7~\mathrm {TeV})$, $2 < y < 2.5$										
$p_T^\mathrm{min}$	$p_T^\mathrm{max}$	$y^\mathrm{min}$	$y^\mathrm{max}$	Central	Scales$-$	Scales$+$	Mass$-$	Mass$+$	PDFs$-$	PDFs$+$
0.00	0.25	2.00	2.50	1.3660	1.3319	1.8150	1.3417	1.3750	1.0613	1.6708
0.25	0.50	2.00	2.50	1.3714	1.3341	1.8565	1.3501	1.3809	1.0942	1.6485
0.50	0.75	2.00	2.50	1.3785	1.3559	1.9302	1.3630	1.3900	1.1222	1.6348
0.75	1.00	2.00	2.50	1.3876	1.3834	1.9488	1.3737	1.3991	1.1369	1.6384
1.00	1.25	2.00	2.50	1.3978	1.3967	1.6868	1.3833	1.4102	1.1516	1.6440
1.25	1.50	2.00	2.50	1.4119	1.4109	1.4738	1.3957	1.4248	1.1817	1.6421
1.50	1.75	2.00	2.50	1.4296	1.3416	1.5023	1.4126	1.4433	1.2274	1.6317
1.75	2.00	2.00	2.50	1.4495	1.3170	1.5304	1.4340	1.4629	1.2816	1.6174
2.00	2.25	2.00	2.50	1.4711	1.3201	1.5579	1.4531	1.4840	1.3364	1.6058
2.25	2.50	2.00	2.50	1.4947	1.3364	1.5838	1.4842	1.5066	1.3883	1.6011
2.50	2.75	2.00	2.50	1.5179	1.3586	1.6083	1.5075	1.5301	1.4345	1.6014
2.75	3.00	2.00	2.50	1.5420	1.3820	1.6331	1.5254	1.5532	1.4760	1.6079
3.00	4.00	2.00	2.50	1.5925	1.4402	1.6836	1.5800	1.6027	1.5504	1.6346
4.00	5.00	2.00	2.50	1.6765	1.5376	1.7642	1.6636	1.6859	1.6531	1.6999
5.00	6.00	2.00	2.50	1.7532	1.6257	1.8369	1.7418	1.7619	1.7356	1.7707
6.00	7.00	2.00	2.50	1.8222	1.7040	1.9030	1.8117	1.8306	1.8070	1.8375
7.00	8.00	2.00	2.50	1.8863	1.7774	1.9629	1.8762	1.8932	1.8722	1.9004
8.00	9.00	2.00	2.50	1.9457	1.8412	2.0201	1.9346	1.9521	1.9325	1.9590
9.00	10.00	2.00	2.50	2.0020	1.9027	2.0747	1.9912	2.0083	1.9888	2.0151
10.00	11.00	2.00	2.50	2.0556	1.9605	2.1263	2.0463	2.0620	2.0423	2.0688
11.00	12.00	2.00	2.50	2.1075	2.0162	2.1773	2.0984	2.1136	2.0940	2.1211
12.00	13.00	2.00	2.50	2.1583	2.0697	2.2280	2.1488	2.1644	2.1443	2.1723
13.00	14.00	2.00	2.50	2.2072	2.1211	2.2765	2.1978	2.2142	2.1927	2.2217
14.00	15.00	2.00	2.50	2.2565	2.1726	2.3256	2.2476	2.2623	2.2414	2.2716
15.00	16.00	2.00	2.50	2.3040	2.2226	2.3726	2.2972	2.3107	2.2885	2.3195
16.00	17.00	2.00	2.50	2.3512	2.2720	2.4210	2.3493	2.3566	2.3351	2.3672
17.00	18.00	2.00	2.50	2.3986	2.3209	2.4681	2.3979	2.4039	2.3820	2.4152
18.00	19.00	2.00	2.50	2.4449	2.3679	2.5140	2.4438	2.4502	2.4278	2.4619
19.00	20.00	2.00	2.50	2.4914	2.4154	2.5609	2.4863	2.4960	2.4738	2.5090
20.00	21.00	2.00	2.50	2.5362	2.4624	2.6064	2.5285	2.5425	2.5181	2.5542
21.00	22.00	2.00	2.50	2.5830	2.5093	2.6531	2.5714	2.5871	2.5644	2.6015
22.00	23.00	2.00	2.50	2.6287	2.5545	2.6986	2.6146	2.6327	2.6098	2.6477
23.00	24.00	2.00	2.50	2.6731	2.6004	2.7451	2.6561	2.6781	2.6537	2.6926
24.00	25.00	2.00	2.50	2.7184	2.6448	2.7896	2.6982	2.7222	2.6986	2.7382
25.00	26.00	2.00	2.50	2.7622	2.6904	2.8346	2.7417	2.7668	2.7420	2.7824
26.00	27.00	2.00	2.50	2.8085	2.7354	2.8792	2.7853	2.8127	2.7878	2.8291
27.00	28.00	2.00	2.50	2.8523	2.7818	2.9260	2.8298	2.8576	2.8312	2.8734
28.00	29.00	2.00	2.50	2.8970	2.8262	2.9716	2.8756	2.9013	2.8754	2.9187
29.00	30.00	2.00	2.50	2.9408	2.8710	3.0169	2.9222	2.9468	2.9186	2.9630

**Table:**

FONLL $pp\rightarrow D^+ + X$, $\sigma ~(13~\mathrm {TeV})/\sigma ~(7~\mathrm {TeV})$, $2.5 < y < 3$										
$p_T^\mathrm{min}$	$p_T^\mathrm{max}$	$y^\mathrm{min}$	$y^\mathrm{max}$	Central	Scales$-$	Scales$+$	Mass$-$	Mass$+$	PDFs$-$	PDFs$+$
0.00	0.25	2.50	3.00	1.3538	1.3369	1.4208	1.3078	1.3816	1.0762	1.6314
0.25	0.50	2.50	3.00	1.3678	1.3473	1.4312	1.3264	1.3917	1.1138	1.6218
0.50	0.75	2.50	3.00	1.3849	1.3678	1.4406	1.3535	1.4064	1.1612	1.6086
0.75	1.00	2.50	3.00	1.4038	1.3957	1.4544	1.3778	1.4250	1.2009	1.6067
1.00	1.25	2.50	3.00	1.4250	1.4201	1.4800	1.3997	1.4448	1.2299	1.6200
1.25	1.50	2.50	3.00	1.4468	1.3960	1.5141	1.4261	1.4657	1.2542	1.6395
1.50	1.75	2.50	3.00	1.4710	1.3692	1.5464	1.4502	1.4885	1.2834	1.6587
1.75	2.00	2.50	3.00	1.4960	1.3681	1.5771	1.4752	1.5127	1.3187	1.6732
2.00	2.25	2.50	3.00	1.5216	1.3806	1.6072	1.5052	1.5371	1.3593	1.6838
2.25	2.50	2.50	3.00	1.5468	1.4010	1.6349	1.5341	1.5621	1.4019	1.6917
2.50	2.75	2.50	3.00	1.5730	1.4238	1.6624	1.5587	1.5859	1.4459	1.7001
2.75	3.00	2.50	3.00	1.5979	1.4481	1.6882	1.5808	1.6105	1.4880	1.7079
3.00	4.00	2.50	3.00	1.6519	1.5055	1.7438	1.6368	1.6644	1.5732	1.7306
4.00	5.00	2.50	3.00	1.7433	1.6042	1.8330	1.7299	1.7543	1.7000	1.7865
5.00	6.00	2.50	3.00	1.8290	1.6977	1.9162	1.8156	1.8393	1.8017	1.8562
6.00	7.00	2.50	3.00	1.9091	1.7855	1.9939	1.8955	1.9193	1.8882	1.9299
7.00	8.00	2.50	3.00	1.9846	1.8690	2.0680	1.9714	1.9942	1.9660	2.0031
8.00	9.00	2.50	3.00	2.0577	1.9452	2.1395	2.0443	2.0668	2.0395	2.0758
9.00	10.00	2.50	3.00	2.1298	2.0219	2.2096	2.1163	2.1380	2.1115	2.1481
10.00	11.00	2.50	3.00	2.1979	2.0954	2.2790	2.1852	2.2073	2.1791	2.2166
11.00	12.00	2.50	3.00	2.2662	2.1678	2.3463	2.2536	2.2750	2.2469	2.2855
12.00	13.00	2.50	3.00	2.3338	2.2375	2.4142	2.3205	2.3427	2.3140	2.3537
13.00	14.00	2.50	3.00	2.4005	2.3069	2.4807	2.3883	2.4089	2.3801	2.4209
14.00	15.00	2.50	3.00	2.4666	2.3751	2.5481	2.4542	2.4746	2.4456	2.4876
15.00	16.00	2.50	3.00	2.5322	2.4419	2.6124	2.5180	2.5395	2.5105	2.5539
16.00	17.00	2.50	3.00	2.5967	2.5082	2.6792	2.5817	2.6051	2.5746	2.6188
17.00	18.00	2.50	3.00	2.6624	2.5745	2.7452	2.6474	2.6706	2.6397	2.6852
18.00	19.00	2.50	3.00	2.7284	2.6415	2.8116	2.7168	2.7362	2.7049	2.7519
19.00	20.00	2.50	3.00	2.7932	2.7057	2.8777	2.7871	2.8025	2.7689	2.8174
20.00	21.00	2.50	3.00	2.8578	2.7720	2.9439	2.8549	2.8663	2.8327	2.8830
21.00	22.00	2.50	3.00	2.9233	2.8374	3.0102	2.9162	2.9321	2.8972	2.9493
22.00	23.00	2.50	3.00	2.9898	2.9039	3.0786	2.9719	2.9972	2.9627	3.0169
23.00	24.00	2.50	3.00	3.0559	2.9695	3.1454	3.0261	3.0622	3.0277	3.0841
24.00	25.00	2.50	3.00	3.1210	3.0363	3.2137	3.0778	3.1305	3.0917	3.1503
25.00	26.00	2.50	3.00	3.1874	3.1014	3.2795	3.1338	3.1950	3.1568	3.2179
26.00	27.00	2.50	3.00	3.2524	3.1676	3.3476	3.1932	3.2619	3.2205	3.2843
27.00	28.00	2.50	3.00	3.3192	3.2349	3.4152	3.2593	3.3295	3.2858	3.3525
28.00	29.00	2.50	3.00	3.3884	3.3033	3.4851	3.3315	3.3981	3.3535	3.4234
29.00	30.00	2.50	3.00	3.4547	3.3690	3.5529	3.4072	3.4655	3.4183	3.4911

**Table:**

FONLL $pp\rightarrow D^+ + X$, $\sigma ~(13~\mathrm {TeV})/\sigma ~(7~\mathrm {TeV})$, $3 < y < 3.5$										
$p_T^\mathrm{min}$	$p_T^\mathrm{max}$	$y^\mathrm{min}$	$y^\mathrm{max}$	Central	Scales$-$	Scales$+$	Mass$-$	Mass$+$	PDFs$-$	PDFs$+$
0.00	0.25	3.00	3.50	1.3693	1.3040	1.4264	1.3123	1.4076	1.0928	1.6458
0.25	0.50	3.00	3.50	1.3876	1.3130	1.4363	1.3368	1.4224	1.1333	1.6419
0.50	0.75	3.00	3.50	1.4110	1.3290	1.4585	1.3692	1.4412	1.1828	1.6392
0.75	1.00	3.00	3.50	1.4378	1.3584	1.4905	1.4011	1.4650	1.2354	1.6401
1.00	1.25	3.00	3.50	1.4656	1.3918	1.5268	1.4346	1.4886	1.2818	1.6495
1.25	1.50	3.00	3.50	1.4935	1.4070	1.5641	1.4675	1.5156	1.3186	1.6684
1.50	1.75	3.00	3.50	1.5221	1.4088	1.6010	1.4986	1.5439	1.3517	1.6926
1.75	2.00	3.00	3.50	1.5522	1.4197	1.6362	1.5272	1.5711	1.3854	1.7191
2.00	2.25	3.00	3.50	1.5821	1.4397	1.6698	1.5635	1.6005	1.4202	1.7441
2.25	2.50	3.00	3.50	1.6108	1.4639	1.7038	1.5924	1.6291	1.4560	1.7657
2.50	2.75	3.00	3.50	1.6412	1.4917	1.7347	1.6202	1.6581	1.4954	1.7870
2.75	3.00	3.00	3.50	1.6701	1.5200	1.7653	1.6510	1.6867	1.5344	1.8057
3.00	4.00	3.00	3.50	1.7342	1.5863	1.8292	1.7158	1.7489	1.6220	1.8463
4.00	5.00	3.00	3.50	1.8415	1.7005	1.9361	1.8248	1.8558	1.7653	1.9176
5.00	6.00	3.00	3.50	1.9455	1.8080	2.0398	1.9280	1.9591	1.8932	1.9977
6.00	7.00	3.00	3.50	2.0460	1.9138	2.1390	2.0278	2.0590	2.0074	2.0846
7.00	8.00	3.00	3.50	2.1427	2.0194	2.2369	2.1259	2.1564	2.1110	2.1745
8.00	9.00	3.00	3.50	2.2397	2.1191	2.3329	2.2230	2.2522	2.2110	2.2684
9.00	10.00	3.00	3.50	2.3351	2.2187	2.4299	2.3183	2.3478	2.3077	2.3625
10.00	11.00	3.00	3.50	2.4303	2.3159	2.5261	2.4132	2.4429	2.4031	2.4574
11.00	12.00	3.00	3.50	2.5257	2.4138	2.6223	2.5070	2.5377	2.4983	2.5531
12.00	13.00	3.00	3.50	2.6215	2.5119	2.7204	2.6029	2.6337	2.5935	2.6496
13.00	14.00	3.00	3.50	2.7175	2.6091	2.8179	2.7000	2.7300	2.6884	2.7466
14.00	15.00	3.00	3.50	2.8130	2.7059	2.9180	2.7955	2.8269	2.7827	2.8434
15.00	16.00	3.00	3.50	2.9124	2.8056	3.0166	2.8899	2.9250	2.8805	2.9443
16.00	17.00	3.00	3.50	3.0091	2.9027	3.1166	2.9855	3.0235	2.9754	3.0429
17.00	18.00	3.00	3.50	3.1087	3.0014	3.2173	3.0885	3.1211	3.0728	3.1446
18.00	19.00	3.00	3.50	3.2100	3.1019	3.3203	3.1997	3.2227	3.1718	3.2483
19.00	20.00	3.00	3.50	3.3091	3.2016	3.4241	3.3136	3.3220	3.2682	3.3499
20.00	21.00	3.00	3.50	3.4109	3.3027	3.5263	3.4245	3.4257	3.3672	3.4545
21.00	22.00	3.00	3.50	3.5099	3.4033	3.6311	3.5249	3.5268	3.4635	3.5563
22.00	23.00	3.00	3.50	3.6120	3.5030	3.7339	3.6217	3.6279	3.5628	3.6612
23.00	24.00	3.00	3.50	3.7134	3.6038	3.8402	3.7094	3.7308	3.6614	3.7655
24.00	25.00	3.00	3.50	3.8185	3.7081	3.9462	3.7996	3.8358	3.7629	3.8741
25.00	26.00	3.00	3.50	3.9234	3.8092	4.0526	3.8875	3.9400	3.8645	3.9822
26.00	27.00	3.00	3.50	4.0254	3.9105	4.1618	3.9766	4.0437	3.9629	4.0880
27.00	28.00	3.00	3.50	4.1309	4.0124	4.2699	4.0703	4.1499	4.0648	4.1971
28.00	29.00	3.00	3.50	4.2399	4.1191	4.3811	4.1711	4.2574	4.1699	4.3100
29.00	30.00	3.00	3.50	4.3505	4.2263	4.4940	4.2810	4.3675	4.2763	4.4246

**Table:**

FONLL $pp\rightarrow D^+ + X$, $\sigma ~(13~\mathrm {TeV})/\sigma ~(7~\mathrm {TeV})$, $3.5 < y < 4$										
$p_T^\mathrm{min}$	$p_T^\mathrm{max}$	$y^\mathrm{min}$	$y^\mathrm{max}$	Central	Scales$-$	Scales$+$	Mass$-$	Mass$+$	PDFs$-$	PDFs$+$
0.00	0.25	3.50	4.00	1.4119	1.3346	1.4761	1.3506	1.4542	1.1266	1.6972
0.25	0.50	3.50	4.00	1.4300	1.3439	1.4888	1.3741	1.4698	1.1674	1.6926
0.50	0.75	3.50	4.00	1.4574	1.3601	1.5130	1.4075	1.4937	1.2183	1.6965
0.75	1.00	3.50	4.00	1.4898	1.3882	1.5489	1.4497	1.5219	1.2753	1.7043
1.00	1.25	3.50	4.00	1.5230	1.4241	1.5899	1.4907	1.5516	1.3292	1.7169
1.25	1.50	3.50	4.00	1.5574	1.4553	1.6328	1.5269	1.5847	1.3773	1.7375
1.50	1.75	3.50	4.00	1.5936	1.4712	1.6756	1.5629	1.6191	1.4221	1.7651
1.75	2.00	3.50	4.00	1.6292	1.4932	1.7162	1.6039	1.6538	1.4623	1.7962
2.00	2.25	3.50	4.00	1.6658	1.5207	1.7576	1.6402	1.6880	1.5017	1.8298
2.25	2.50	3.50	4.00	1.7009	1.5519	1.7962	1.6747	1.7226	1.5400	1.8619
2.50	2.75	3.50	4.00	1.7375	1.5840	1.8347	1.7121	1.7582	1.5806	1.8944
2.75	3.00	3.50	4.00	1.7725	1.6175	1.8721	1.7478	1.7929	1.6205	1.9245
3.00	4.00	3.50	4.00	1.8520	1.6964	1.9536	1.8267	1.8713	1.7146	1.9893
4.00	5.00	3.50	4.00	1.9909	1.8384	2.0967	1.9670	2.0097	1.8812	2.1007
5.00	6.00	3.50	4.00	2.1286	1.9817	2.2343	2.1048	2.1468	2.0427	2.2146
6.00	7.00	3.50	4.00	2.2640	2.1224	2.3716	2.2398	2.2822	2.1959	2.3321
7.00	8.00	3.50	4.00	2.4001	2.2623	2.5118	2.3747	2.4177	2.3442	2.4560
8.00	9.00	3.50	4.00	2.5382	2.4009	2.6527	2.5124	2.5566	2.4900	2.5863
9.00	10.00	3.50	4.00	2.6784	2.5417	2.7980	2.6517	2.6976	2.6343	2.7225
10.00	11.00	3.50	4.00	2.8230	2.6847	2.9443	2.7940	2.8410	2.7803	2.8658
11.00	12.00	3.50	4.00	2.9672	2.8297	3.0947	2.9385	2.9863	2.9232	3.0111
12.00	13.00	3.50	4.00	3.1128	2.9746	3.2456	3.0829	3.1339	3.0662	3.1593
13.00	14.00	3.50	4.00	3.2626	3.1222	3.3974	3.2311	3.2826	3.2122	3.3129
14.00	15.00	3.50	4.00	3.4105	3.2690	3.5505	3.3792	3.4318	3.3559	3.4651
15.00	16.00	3.50	4.00	3.5609	3.4171	3.7082	3.5266	3.5840	3.5017	3.6201
16.00	17.00	3.50	4.00	3.7148	3.5681	3.8651	3.6782	3.7384	3.6503	3.7793
17.00	18.00	3.50	4.00	3.8705	3.7182	4.0255	3.8327	3.8919	3.8004	3.9405
18.00	19.00	3.50	4.00	4.0264	3.8714	4.1890	3.9925	4.0503	3.9506	4.1022
19.00	20.00	3.50	4.00	4.1897	4.0286	4.3555	4.1604	4.2131	4.1076	4.2719
20.00	21.00	3.50	4.00	4.3557	4.1882	4.5273	4.3320	4.3788	4.2665	4.4449
21.00	22.00	3.50	4.00	4.5219	4.3480	4.7036	4.4982	4.5477	4.4249	4.6189
22.00	23.00	3.50	4.00	4.6941	4.5132	4.8858	4.6688	4.7229	4.5883	4.7999
23.00	24.00	3.50	4.00	4.8789	4.6878	5.0777	4.8517	4.9077	4.7628	4.9950
24.00	25.00	3.50	4.00	5.0687	4.8704	5.2825	5.0438	5.1015	4.9406	5.1967
25.00	26.00	3.50	4.00	5.2668	5.0621	5.4913	5.2404	5.2989	5.1263	5.4073
26.00	27.00	3.50	4.00	5.4690	5.2542	5.7074	5.4396	5.4992	5.3145	5.6234
27.00	28.00	3.50	4.00	5.6810	5.4567	5.9367	5.6446	5.7101	5.5107	5.8512
28.00	29.00	3.50	4.00	5.9091	5.6725	6.1796	5.8599	5.9408	5.7209	6.0972
29.00	30.00	3.50	4.00	6.1519	5.8982	6.4438	6.0920	6.1871	5.9437	6.3601

**Table:**

FONLL $pp\rightarrow D^+ + X$, $\sigma ~(13~\mathrm {TeV})/\sigma ~(7~\mathrm {TeV})$, $4 < y < 4.5$										
$p_T^\mathrm{min}$	$p_T^\mathrm{max}$	$y^\mathrm{min}$	$y^\mathrm{max}$	Central	Scales$-$	Scales$+$	Mass$-$	Mass$+$	PDFs$-$	PDFs$+$
0.00	0.25	4.00	4.50	1.4764	1.4013	1.5479	1.4123	1.5257	1.1721	1.7807
0.25	0.50	4.00	4.50	1.4975	1.4111	1.5626	1.4349	1.5432	1.2193	1.7756
0.50	0.75	4.00	4.50	1.5280	1.4282	1.5893	1.4746	1.5700	1.2749	1.7812
0.75	1.00	4.00	4.50	1.5650	1.4569	1.6297	1.5223	1.6038	1.3353	1.7947
1.00	1.25	4.00	4.50	1.6052	1.4958	1.6778	1.5655	1.6414	1.3940	1.8164
1.25	1.50	4.00	4.50	1.6482	1.5357	1.7287	1.6103	1.6814	1.4506	1.8458
1.50	1.75	4.00	4.50	1.6928	1.5618	1.7805	1.6592	1.7238	1.5062	1.8794
1.75	2.00	4.00	4.50	1.7371	1.5947	1.8320	1.7089	1.7673	1.5575	1.9166
2.00	2.25	4.00	4.50	1.7833	1.6310	1.8835	1.7525	1.8129	1.6079	1.9588
2.25	2.50	4.00	4.50	1.8301	1.6719	1.9345	1.7964	1.8589	1.6569	2.0033
2.50	2.75	4.00	4.50	1.8783	1.7155	1.9847	1.8445	1.9059	1.7068	2.0497
2.75	3.00	4.00	4.50	1.9268	1.7595	2.0359	1.8931	1.9534	1.7575	2.0962
3.00	4.00	4.00	4.50	2.0317	1.8617	2.1449	1.9978	2.0581	1.8701	2.1933
4.00	5.00	4.00	4.50	2.2249	2.0555	2.3467	2.1908	2.2518	2.0804	2.3693
5.00	6.00	4.00	4.50	2.4229	2.2518	2.5517	2.3873	2.4495	2.2988	2.5471
6.00	7.00	4.00	4.50	2.6261	2.4534	2.7627	2.5874	2.6536	2.5199	2.7322
7.00	8.00	4.00	4.50	2.8341	2.6601	2.9776	2.7951	2.8640	2.7414	2.9267
8.00	9.00	4.00	4.50	3.0479	2.8712	3.1983	3.0064	3.0778	2.9635	3.1324
9.00	10.00	4.00	4.50	3.2652	3.0844	3.4231	3.2211	3.2935	3.1841	3.3463
10.00	11.00	4.00	4.50	3.4865	3.3001	3.6538	3.4409	3.5159	3.4050	3.5680
11.00	12.00	4.00	4.50	3.7122	3.5198	3.8902	3.6623	3.7421	3.6267	3.7976
12.00	13.00	4.00	4.50	3.9419	3.7449	4.1330	3.8924	3.9768	3.8497	4.0341
13.00	14.00	4.00	4.50	4.1861	3.9775	4.3912	4.1318	4.2206	4.0842	4.2880
14.00	15.00	4.00	4.50	4.4361	4.2161	4.6579	4.3767	4.4737	4.3200	4.5522
15.00	16.00	4.00	4.50	4.7043	4.4661	4.9448	4.6422	4.7448	4.5695	4.8390
16.00	17.00	4.00	4.50	4.9965	4.7401	5.2586	4.9282	5.0350	4.8396	5.1534
17.00	18.00	4.00	4.50	5.2978	5.0180	5.5895	5.2225	5.3423	5.1154	5.4802
18.00	19.00	4.00	4.50	5.6218	5.3046	5.9377	5.5270	5.6729	5.4094	5.8343
19.00	20.00	4.00	4.50	5.9807	5.6363	6.3372	5.8742	6.0463	5.7306	6.2308
20.00	21.00	4.00	4.50	6.3843	6.0026	6.7857	6.2636	6.4522	6.0891	6.6795
21.00	22.00	4.00	4.50	6.8090	6.3860	7.2550	6.6782	6.8789	6.4621	7.1559
22.00	23.00	4.00	4.50	7.2650	6.7952	7.7642	7.1254	7.3405	6.8590	7.6711
23.00	24.00	4.00	4.50	7.7768	7.2502	8.3333	7.6282	7.8653	7.3003	8.2532
24.00	25.00	4.00	4.50	8.3491	7.7525	8.9783	8.1949	8.4510	7.7914	8.9068
25.00	26.00	4.00	4.50	8.9707	8.3089	9.6674	8.8223	9.0938	8.3241	9.6172
26.00	27.00	4.00	4.50	9.6443	8.9062	10.4147	9.4929	9.7709	8.9044	10.3843
27.00	28.00	4.00	4.50	10.3739	9.5536	11.2325	10.2285	10.5085	9.5259	11.2220
28.00	29.00	4.00	4.50	11.1723	10.2538	12.1237	11.0109	11.3198	10.1975	12.1472
29.00	30.00	4.00	4.50	12.0408	11.0141	13.0937	11.8407	12.2099	10.9208	13.1607

**Table:**

FONLL $pp\rightarrow B^+ X$, $d\sigma /d\eta ~(13~\mathrm {TeV})$ [pb], $p_T > 0$, $f(b\rightarrow B^+) = 0.403$							
$\eta$	Central	Scales$-$	Scales$+$	Mass$-$	Mass$+$	PDFs$-$	PDFs$+$
0.00	1.527e+07	9.797e+06	2.144e+07	1.329e+07	1.763e+07	1.479e+07	1.575e+07
0.25	1.544e+07	9.898e+06	2.169e+07	1.344e+07	1.782e+07	1.495e+07	1.592e+07
0.50	1.591e+07	1.018e+07	2.237e+07	1.387e+07	1.837e+07	1.541e+07	1.642e+07
0.75	1.660e+07	1.057e+07	2.335e+07	1.447e+07	1.914e+07	1.606e+07	1.713e+07
1.00	1.735e+07	1.099e+07	2.443e+07	1.514e+07	1.992e+07	1.678e+07	1.785e+07
1.25	1.797e+07	1.137e+07	2.534e+07	1.570e+07	2.068e+07	1.738e+07	1.857e+07
1.50	1.849e+07	1.161e+07	2.609e+07	1.616e+07	2.126e+07	1.786e+07	1.911e+07
1.75	1.878e+07	1.176e+07	2.653e+07	1.642e+07	2.158e+07	1.812e+07	1.943e+07
2.00	1.880e+07	1.176e+07	2.654e+07	1.646e+07	2.159e+07	1.814e+07	1.948e+07
2.25	1.862e+07	1.162e+07	2.637e+07	1.624e+07	2.140e+07	1.786e+07	1.932e+07
2.50	1.820e+07	1.137e+07	2.580e+07	1.590e+07	2.093e+07	1.747e+07	1.892e+07
2.75	1.758e+07	1.098e+07	2.497e+07	1.535e+07	2.024e+07	1.684e+07	1.833e+07
3.00	1.681e+07	1.055e+07	2.391e+07	1.467e+07	1.938e+07	1.604e+07	1.759e+07
3.25	1.592e+07	1.005e+07	2.268e+07	1.386e+07	1.838e+07	1.509e+07	1.675e+07
3.50	1.492e+07	9.487e+06	2.129e+07	1.296e+07	1.727e+07	1.401e+07	1.583e+07
3.75	1.384e+07	8.870e+06	1.979e+07	1.200e+07	1.606e+07	1.292e+07	1.486e+07
4.00	1.270e+07	8.201e+06	1.819e+07	1.097e+07	1.478e+07	1.165e+07	1.385e+07
4.25	1.151e+07	7.500e+06	1.652e+07	9.918e+06	1.344e+07	1.033e+07	1.279e+07
4.50	1.029e+07	6.766e+06	1.481e+07	8.842e+06	1.206e+07	9.003e+06	1.167e+07
4.75	9.072e+06	6.017e+06	1.308e+07	7.762e+06	1.067e+07	7.697e+06	1.053e+07
5.00	7.863e+06	5.263e+06	1.137e+07	6.702e+06	9.289e+06	6.452e+06	9.354e+06
5.25	6.694e+06	4.522e+06	9.712e+06	5.682e+06	7.943e+06	5.287e+06	8.173e+06
5.50	5.586e+06	3.808e+06	8.129e+06	4.719e+06	6.662e+06	4.232e+06	7.004e+06
5.75	4.562e+06	3.137e+06	6.662e+06	3.835e+06	5.469e+06	3.305e+06	5.876e+06
6.00	3.660e+06	2.519e+06	5.332e+06	3.061e+06	4.413e+06	2.509e+06	4.812e+06

**Table:**

FONLL $pp\rightarrow B^+ X$, $d\sigma /d\eta ~(13~\mathrm {TeV})/d\sigma /d\eta ~(7~\mathrm {TeV})$, $p_T > 0$							
$\eta$	Central	Scales$-$	Scales$+$	Mass$-$	Mass$+$	PDFs$-$	PDFs$+$
0.00	1.6527	1.5583	1.7069	1.6388	1.6662	1.6353	1.6701
0.25	1.6527	1.5574	1.7068	1.6388	1.6655	1.6353	1.6701
0.50	1.6509	1.5567	1.7052	1.6372	1.6639	1.6336	1.6683
0.75	1.6492	1.5542	1.7094	1.6410	1.6625	1.6308	1.6675
1.00	1.6535	1.5521	1.7029	1.6346	1.6671	1.6367	1.6703
1.25	1.6490	1.5560	1.7033	1.6344	1.6628	1.6317	1.6664
1.50	1.6512	1.5536	1.7058	1.6370	1.6653	1.6340	1.6684
1.75	1.6557	1.5574	1.7069	1.6394	1.6697	1.6390	1.6723
2.00	1.6616	1.5646	1.7189	1.6461	1.6786	1.6440	1.6791
2.25	1.6751	1.5785	1.7316	1.6599	1.6866	1.6581	1.6922
2.50	1.6910	1.5912	1.7477	1.6752	1.7060	1.6732	1.7088
2.75	1.7110	1.6071	1.7689	1.6952	1.7271	1.6906	1.7314
3.00	1.7376	1.6356	1.7951	1.7215	1.7537	1.7113	1.7639
3.25	1.7713	1.6738	1.8288	1.7546	1.7874	1.7338	1.8088
3.50	1.8120	1.7182	1.8689	1.7948	1.8289	1.7570	1.8671
3.75	1.8602	1.7692	1.9174	1.8428	1.8782	1.7828	1.9376
4.00	1.9178	1.8265	1.9737	1.8990	1.9367	1.8175	2.0182
4.25	1.9847	1.8921	2.0406	1.9635	2.0057	1.8634	2.1060
4.50	2.0613	1.9700	2.1207	2.0388	2.0856	1.9202	2.2025
4.75	2.1520	2.0589	2.2100	2.1269	2.1768	1.9918	2.3122
5.00	2.2563	2.1630	2.3025	2.2271	2.2843	2.0760	2.4365
5.25	2.3617	2.2824	2.4227	2.3434	2.3939	2.1583	2.5652
5.50	2.4679	2.4203	2.5277	2.4341	2.5319	2.2394	2.6965
5.75	2.6523	2.5341	2.7320	2.6329	2.6889	2.4158	2.8888
6.00	2.8710	2.7584	2.9170	2.8280	2.9122	2.6185	3.1235

## Footnotes

1 It is a well-known fact, which we do not need to corroborate further with explicit figures, that charm and bottom production in *pp* collisions is fully dominated by *gg* initial states.

2 In the rest of this work we focus on the 7 TeV data, since it is at this energy that most—if not all—of the charm and bottom production measurements have been reported.

3 While this work was in preparation, Ref. [33] also considered energy ratios in the context of heavy-quark production, while Ref. [34] studied the total charm hadroproduction cross section using two different charm mass schemes and multiple PDF sets.

4 NNLO results for differential distributions of heavy-quark pair production are in principle known [39, 40]. In practice, the calculation of the differential transverse momentum spectra for charm and bottom quarks still requires dedicated numerical studies by the authors of the original NNLO calculations.

5 The situation at a centre-of-mass energy of 7 TeV, not shown, is qualitatively identical.

6 Note that the uncertainty due to the mass value is not shown as it is always totally negligible with respect to the others.
